# Targeting HER2 in patient‐derived xenograft ovarian cancer models sensitizes tumors to chemotherapy

**DOI:** 10.1002/1878-0261.12414

**Published:** 2018-12-21

**Authors:** Faye R. Harris, Piyan Zhang, Lin Yang, Xiaonan Hou, Konstantinos Leventakos, Saravut J. Weroha, George Vasmatzis, Irina V. Kovtun

**Affiliations:** ^1^ Center for Individualized Medicine Mayo Clinic Rochester MN USA; ^2^ Departments of Medical Oncology Mayo Clinic Rochester MN USA; ^3^ Molecular Pharmacology and Experimental Therapeutics Mayo Clinic Rochester MN USA; ^4^ Molecular Medicine Mayo Clinic Rochester MN USA

**Keywords:** ERBB pathway, HER2, mate‐pair next generation sequencing, ovarian cancer, patient‐derived xenografts

## Abstract

Ovarian cancer is the most lethal gynecologic malignancy. About 75% of ovarian cancer patients relapse and/or develop chemo‐resistant disease after initial response to standard‐of‐care treatment with platinum‐based therapies. HER2 amplifications and overexpression in ovarian cancer are reported to vary, and responses to HER2 inhibitors have been poor. Next generation sequencing technologies in conjunction with testing using patient‐derived xenografts (PDX) allow validation of personalized treatments. Using a whole‐genome mate‐pair next generation sequencing (MPseq) protocol, we identified several high grade serous ovarian cancers (HGS‐OC) with DNA alterations in genes encoding members of the ERBB2 pathway. The efficiency of anti‐HER2 therapy was tested in three different PDX lines with the identified alterations and high levels of HER2 protein expression. Treatment responses to pertuzumab or pertuzumab/trastuzumab were compared in each PDX line WITH standard carboplatin and paclitaxel combination treatment. In all three PDX models, HER2‐targeted therapy resulted in significant inhibition of tumor growth compared with untreated controls. However, the responses in each case were inferior to those to chemotherapy, even for chemo‐resistant lines. When chemotherapy and HER2‐targeted therapy were administered together, a significant regression of tumor was observed after 6 weeks of treatment compared with chemotherapy alone. Post‐treatment analysis of these tissues revealed that inhibition of the ERBB2 pathway occurred at the level of phosphorylation and expression of downstream targets. In conclusion, while targeting of presumably activated ERBB2 pathway alone in HGS‐OC results in a modest treatment benefit, a combination therapy including both chemotherapy drugs and HER2 inhibitors provides a far better response. Further studies are needed to address development of recurrence and sensitivity of recurrent disease to HER2‐targeted therapy.

**Abbreviations**
AKTAKT: serine/threonine kinase, protein kinase B
CNV: copy number variation
EGFR: epidermal growth factor receptor
ERBB: avian erythroblastosis oncogene B
ERK: extracellular receptor kinase
FFPE: formalin‐fixed, paraffin‐embedded
HER2: human epidermal growth factor receptor 2
HGS‐OC: high grade serous ovarian cancer
IHC: immunohistochemistry
MPseq: mate‐pair next generation sequencing
NRG1: neuroregulin 1
PDX: patient‐derived xenografts
PZ: pertuzumab
qPCR: quantitative polymerase chain reaction
RNAseq: RNA sequencing
TZ: trastuzumab

## 1 Introduction

High grade serous ovarian cancer (HGS‐OC) is one of the deadliest cancers, with detection most commonly occurring at late stages when it has already spread to the peritoneum. The majority of patients undergo debulking surgery and initially respond to the standard treatment of taxane and platinum‐based drug combination, but later progress and develop chemo‐resistant disease. More therapies are needed to tackle OC and improve patient survival. Recent efforts in oncology have focused on testing targeted therapies chosen based on a molecular characterization of individual tumors. Among these are inhibitors of HER receptors, primarily EGFR and HER2.

Receptor tyrosine‐protein kinase HER2 belongs to an ERBB signaling pathway which includes structurally related receptor kinases EGFR (HER1), HER3 and HER4 and their ligands (Hynes and Lane, 2005). HER2 is a receptor with no known natural ligand that upon dimerization with EGFR, HER3 or HER4 activates RAS/ERK and PI3K/AKT signaling pathways (Arteaga and Engelman, 2014; Yakes *et al*., 2002) and promotes cell growth, cell migration and invasion (Appert‐Collin *et al*., 2015; Hynes and Lane, 2005). When overexpressed, HER2 is believed to increase the affinity of EGF and other ligands to their corresponding receptors (Sliwkowski *et al*., 1994; Wada *et al*., 1990). Increased levels of HER2 have been shown to affect rates of degradation for HER2‐containing heterodimers, thus prolonging the pathway stimulation (Huang *et al*., 1999; Parakh *et al*., 2017; Sak *et al*., 2013).

Human epidermal growth factor receptor 2 is known to play an important role in the progression of aggressive types of breast cancer, where the corresponding ERBB2 gene is shown to be amplified in 15–20% of cases (Di Cosimo and Baselga, 2010). The proteins of the ERBB pathway, HER2 in particular, have also been implicated in driving HGS‐OC (Lafky *et al*., 2008; Lassus *et al*., 2006). About 11% of OCs are thought to harbor ERBB2 amplifications (Reibenwein and Krainer, 2008) with overexpression of HER2 protein, estimated to be 21–38% in HGS‐OC (Berchuck *et al*., 1990; Fujimura *et al*., 2002; Jafri and Rizvi, 2017). Separate analysis of stage I and stage III tumors, however, revealed a frequency of HER2 amplification of 17 and 83%, respectively, thus suggesting that amplification (and possibly corresponding overexpression) marks a more aggressive and advanced phenotype (Afify *et al*., 1999; Shang *et al*., 2017).

Several studies have reported an association of the level of HER2 expression and/or amplification with rates of recurrence (Berchuck *et al*., 1990; Fujimura *et al*., 2002), and worse prognosis in OC (Lassus *et al*., 2006; Momeny *et al*., 2017; Nielsen *et al*., 2004; Wang *et al*., 2017). *In vitro*, OC cells overexpressing HER2 protein demonstrated a more aggressive phenotype, and HER2 knockdown has been shown to result in growth inhibition (Montero *et al*., 2015; Yang *et al*., 2004). Whereas some studies have reported an inverse correlation between the expression level of HER2 and the outcome in OC patients (Wang *et al*., 2017), others have found no correlation between levels of HER2 and survival (Lee *et al*., 2005). Although extensive data have been collected on responses to HER2 inhibitors in OC, both in preclinical models and clinical trials, the clinical significance of HER2 expression in OC remains controversial.

Human epidermal growth factor receptor 2 inhibitors are effective in treating breast cancer patients when tumors express the HER2 protein. Several generations of HER2 inhibitors have been developed over the last two decades and are in current clinical use to target different functions of HER2 homo‐ and heterodimers (Parakh *et al*., 2017). Pertuzumab (PZ) and trastuzumab (TZ) are two different monoclonal antibodies directed against the extracellular domains II and IV of HER2, respectively (Adams *et al*., 2006; Albanell and Baselga, 1999; Herbst *et al*., 2007). TZ has been shown to have efficacy in treating HER2‐positive breast cancer as a monotherapy as well as when combined with chemotherapy (Slamon *et al*., 2001; Vogel *et al*., 2002; Vu and Claret, 2012). It is believed to affect tumor growth by several different mechanisms which include the induction of HER2 degradation (Klapper *et al*., 2000), induction of cellular cytotoxicity through recruitment of natural killer cells and cytotoxic proteins (Arnould *et al*., 2006; Clynes *et al*., 2000), and inhibition of the PI3K/AKT pathway (Nagata *et al*., 2004; Zhang *et al*., 2011). Although discovery of TZ has improved the survival of breast cancer patients with HER2‐positive disease, development of resistance has also been noted (Zhang *et al*., 2011). Patients that progress on TZ can benefit from the addition of PZ, an antibody which blocks dimerization of HER2 (Adams *et al*., 2006; Herbst *et al*., 2007). TZ/PZ combination therapy in breast cancer resulted in a clinical benefit rate of 50% and longer progression‐free survival (median of 5.5 months). PZ alone, however, showed only limited benefit (Cortes *et al*., 2012).

In HGS‐OC, the efficacy of antibodies targeting HER2 has been tested in both preclinical studies and clinical trials. Analysis of molecular responses to TZ and PZ, administered separately or in combination in the SKOV3 xenograft model for OC, revealed that the two antibodies inhibit different molecular pathways implicated in HER2 action (Sims *et al*., 2012). TZ appears to affect shedding of the HER2 extracellular domain, in a process known to be associated with disease progression (Sims *et al*., 2012). While pERK signaling is inhibited by both treatments, pAKT signaling can only be inhibited by PZ. In a xenograft model of ovarian clear cell adenocarcinoma consisting of RMG‐1 cells overexpressing HER2, TZ was shown to inhibit cell growth in a dose‐dependent manner, and the survival of TZ‐treated mice was longer than that of the control group (Fujimura *et al*., 2002). The same study reported that the extent of the inhibitory effect of TZ in various cell models for OC was dependent on the HER2 expression level.

The antibody‐drug conjugate TZ‐emtansine (Lambert and Chari, 2014) showed superior responses compared with TZ, PZ or lapatinib in all tested OC cell lines (both sensitive and insensitive to knockdown of HER2) *in vitro* as well as in xenograft models *in vivo* (Montero *et al*., 2015). Unlike TZ or PZ, which only inhibited tumor growth in a xenograft model, TZ‐emtansine caused tumor regression, thus suggesting that a combination of HER2 antibodies with chemotherapeutic agents might be more effective in treating OC than would single agent therapy (Montero *et al*., 2015). Similarly, dacomitinib, a second generation small molecule pan‐ERBB inhibitor known to block the kinase domains of EGFR, HER2 and HER4 (Engelman *et al*., 2007; Gonzales *et al*., 2008), was reportedly effective in suppressing the growth and invasive capacity of chemo‐resistant OC cell lines *in vitro* where monoclonal antibodies to EGFR, HER2, or HER3 were largely ineffective (Momeny *et al*., 2017). Targeting of another ERBB family receptor HER3 with its specific monoclonal antibody was also reported to result in tumor growth inhibition in an OC xenograft model using OVCAR8 cells (Sheng *et al*., 2010).

Although the inhibition of HER2 using TZ or PZ in model systems showed promising responses, the outcomes of clinical trials were quite disappointing (Reibenwein and Krainer, 2008). A 7% response rate to TZ was reported by Bookman *et al*. (2003) in patients with OC who were selected based on 2^+^/3^+^  expression levels of HER2 evaluated by immunohistochemistry (IHC). Stabilization of disease was noted in 39% of patients (Bookman *et al*., 2003). In a later study, the efficacy of PZ as a single agent in patients with relapsed OC was evaluated. Partial response was noted in 4.3% of patients, and 6.8% had stable disease (Gordon *et al*., 2006). Molecular analysis of HER2 activation, i.e. its phosphorylation status (pHER2), revealed significantly longer survival (20.9 weeks) for patients with pHER2^+^ status as compared with pHER2^–^ (5.8 weeks) (Gordon *et al*., 2006). PZ has been demonstrated to augment response to gemcitabine in patients who developed platinum‐resistant disease. A response rate of 13.8% versus 4.6% in combination treatment compared with gemcitabine alone was observed (Makhija *et al*., 2010). Similarly, in a phase III clinical trial, slightly longer progression‐free survival was observed in patients with platinum‐resistant disease who received PZ in combination with either paclitaxel or gemcitabine (Kurzeder *et al*., 2016). More refined molecular characterization of ovarian tumors may be needed to identify subgroups of patients that may benefit from an addition of ERBB‐targeted therapies.

In this study, using a whole‐genome mate‐pair next generation sequencing (MPseq) protocol we identified several HGS‐OC tumors with DNA alterations at genes of the ERBB2 pathway. In each tumor these changes constituted top‐hits for potential targeted therapy. One tumor harbored a duplication of ERBB2 and high expression of HER2 protein. A second harbored an amplification of the NRG3 gene, a known ligand for the HER4 receptor. The third tumor had a fusion at the NRG1 gene, a ligand for HER3 and HER4 receptors that was highly expressed at the protein level. We compared responses to anti‐HER2 therapy in PDX models associated with each of these tumors. The landscape of DNA alterations in the primary tumors and their derivative tumors propagated in mice revealed a striking similarity. In one case, a few additional changes were identified in PDX, consistent with the concept of tumor evolution upon growth. HER2‐targeted therapy alone resulted in a significant inhibition of tumor growth compared with untreated controls in all three tested PDXs. The responses to each were inferior to chemotherapy, even in a chemo‐resistant case, suggesting that anti‐HER2 treatment in OC as a single therapy is not effective. There was an extra benefit when HER2‐targeted therapy was administered together with chemotherapy.

## 2 Materials and methods

### 2.1 Antibodies and drugs

Anti‐HER2 (cat. #2242), anti‐HER3 (cat. #12708), anti‐pHER2 (cat. #2243), anti‐EGFR (cat. #2232), anti‐pEGFR (cat. #3777), anti‐AKT (cat. #9272), anti‐pAKT (cat. #13038), anti‐ERK (cat. #4695), and anti‐pERK (cat. #4370) were from Cell Signaling Inc. (Beverly, MA, USA). Anti‐NRG was from ThermoFisher Scientific (Waltham, MA, USA; cat. PA5‐13204), and anti‐GAPDH from Santa Cruz Biotechnology, Dallas, TX, USA (cat. Sc‐365062). Carboplatin (cat. #61703‐360‐18) and paclitaxel (cat# 55390‐304‐05) were from Novaplus (Irving, TX, USA). Pertuzumab and trastuzumab were from Genentech, San Francisco, CA, USA (clinical grade) and lapatinib was purchased from Sigma‐Aldrich, St. Louis, MO, USA (cat. CDS022971).

### 2.2 PDX maintenance and treatment

Fresh tissues from consenting, treatment‐naive patients with OC were collected at the time of primary debulking surgery in accordance with the Mayo Clinic Institutional Review Board through the Mayo Clinic Ovarian Tumor Repository. Initial written consent was obtained from all patients. The study methodologies conformed to the standards set by the Declaration of Helsinki. Tumor grafts were developed as previously described (Weroha *et al*., 2014) by intraperitoneal (IP) injection into female SCID beige mice (C.B.‐17/IcrHsd‐*Prkdcscid Lystbg*; ENVIGO) in accordance with the Mayo Clinic Institutional Animal Care and Use Committee. Briefly, ~0.3 mL of minced fresh patient tumor was mixed 1 : 1 with McCoy's media in a 1‐mL syringe and injected IP into 6‐ to 8‐week‐old mice. No enzymatic or mechanical tumor dissociation was performed. Mice were monitored by routine palpation for engraftment, and tumors were harvested when moribund. Bodyweight and the general condition of mice were assessed at least twice a week except for animals under therapy, which were monitored daily. Tumors were classified as chemo‐resistant if progression was observed during or within 6 months of completing chemotherapy.

**Figure 1:**
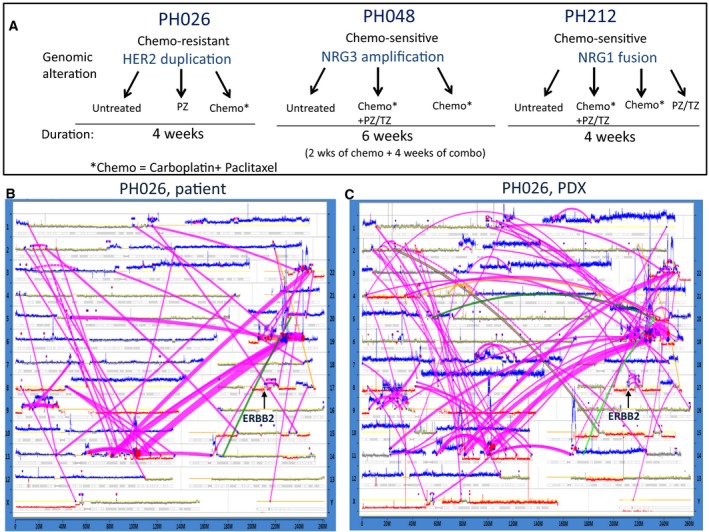
Genomic characterization of ovarian tumors using MPseq. (A) Schematic representation of strategy of the treatment for three different PDX models. The clinical properties and molecular alterations of each tumor are indicated. (B,C) Genome plots for the patient's tumor (B) and matching PDX (C) showing the landscape of structural alterations and copy number changes as detected by MP. The *X*‐axis spans the length of the chromosome with chromosome position number shown. Each chromosome is indicated on the right and left *Y*‐axis. The height of the horizontal traces for each chromosome indicates the number of reads detected for 30‐k base pair windows. DNA copy numbers are indicated by color, with gray representing the normal 2 N copy state, red corresponding to deletions, and blue to gains. Connecting magenta lines correspond to chromosomal rearrangements. The widths of the lines correlate to the number of associated mate‐pair reads. Alterations at ERBB2 locus are depicted.

Treatments were started when palpated tumors reached 0.5–1 cm^2^. For PDX PH212, which was the model for ascites, treatment started 1 week after injection. Drugs were withheld if weight dropped 20% or more from initial weight. Chemotherapy consists of carboplatin (51 mg·kg^−1^) and paclitaxel (15 mg·kg^−1^) administered intraperitoneally (IP) once a week. No ascites were observed in the other two tested PDX lines, PDX026 and PDX048. For PH026 all therapies were administered for 4 weeks, with PZ injected IP 3 times a week at 20 mg·kg^−1^ (Fig. 1A). For PH048, chemotherapy was administered for 6 weeks with targeted therapy starting at week 2 of chemotherapy and continuing for 4 weeks. PZ/TZ were administered together at 20 mg·kg^−1^ IP three times a week. For PH212, all therapies were administered for 4 weeks (Fig. 1A). PZ/TZ were administered as for PH048 (above), and lapatinib was given 5 times a week at 150 mg·kg^−1^ by oral gavage. The tumor size was assessed weekly by ultrasound; three measurements per session for each animal were made and averaged.

### 2.3 Next generation sequencing

#### 2.3.1 Mate‐pair

About 1000 ng of DNA and Illumina Nextera Mate Pair Library Prep Kit (Illumina, San Diego, CA, USA; FC‐132‐1001) were used to make MPseq libraries, which were sequenced as two samples per lane on the Illumina Hi‐Seq 2000. A set of algorithms was used to detect large chromosomal aberrations (deletions, amplifications, inversions, and translocations) as described earlier (Kovtun *et al*., 2015). Briefly, the read‐to‐reference–genome‐mapping algorithm consisted of (a) indexing the reference genome; (b) finding all possible mapping positions of both reads; and (c) aligning of the read pairs to find the optimal map position of the fragment. The protocol allows sequencing of the ends of large fragments of genomic DNA (2.5–5 kb), thus effectively covering breakpoints by 30× on average. Both bridged and base pair coverage were calculated. Breakpoints covered by at least five mate‐pairs in each sample were collected for further analysis. Unmapped read‐pairs (~3–6% of all read‐pairs) were removed from the data. Filters, based on homology scores calculated during mapping, were applied to eliminate false positives from the selection of events. Copy number variations (CNV) were identified by analyzing frequency distributions of window counts of mapped reads across the reference genome as previously described (Smadbeck *et al*., 2018). Deletion and amplification peaks and valleys of the frequency distribution were determined by finding the spots where the discrete derivative of the distribution would cross zero. The dominant mode was determined by finding the highest peak of that distribution which was also the maximum of the density function. The nearest left minimum was considered a threshold below which deletions were called. Amplifications were called by finding the neighboring minimum on the right of the highest peak.

#### 2.3.2 RNA sequencing

RNA was isolated from frozen tumors or ascites pellets using the RNeasy Plus Mini Kit (Qiagen 74134, Valencia, CA, USA) or (Qiagen 74104) for FFPE sample. RNA libraries were prepared according to the manufacturer's instructions for the TruSeq® RNA Access Library Prep Kit (Illumina). Briefly, coding regions of the transcriptome are captured by pooling four of the cDNA libraries at 200 ng each. Libraries were sequenced at ~100 million reads per sample (3 samples/lane) following Illumina's standard protocol using the Illumina cBot and HiSeq 3000/4000 PE Cluster Kit. The flow cells were sequenced as 100 × 2 paired end reads on an Illumina HiSeq 4000 using HiSeq 3000/4000 sequencing kit and hcs v3.3.20 collection software. Base‐calling was performed using illumina rta version 2.5.2 (Illumina, San Diego, CA, USA).

The analysis was performed using a pipeline at the Bioinformatics Core at Mayo Clinic. Briefly, raw reads from PDX samples were first processed with xenome (version 1.0.1) (Conway *et al*., 2012) to be classified as ‘mouse’ or ‘human’. Only the reads classified as ‘graft’, ‘ambiguous’ or ‘both’ were included in the downstream analysis. ‘Human’ portion of the PDX and donor patient samples were then processed with map‐rseq version 3.0.2 (Kalari *et al*., 2014). Alignment and mapping of reads was performed using STAR aligner (Dobin *et al*., 2013) against the hg38 reference genome. The gene and exon counts were generated by FeatureCounts (Liao *et al*., 2014) using the gene definitions files from Ensembl GRCh38.78. All samples passed quality control according to RSeqQC criteria (Wang *et al*., 2012) as well as additional checks (Liu *et al*., 2017). Fusion events were detected using star‐fusion (version 0.8.0).

### 2.4 DNA fingerprinting

Genomic DNA isolated from the original tumor and tumor ascites from PDX was analyzed for the presence of 34 single‐nucleotide polymorphisms (SNP) that have been reported to maximize the probability of obtaining distinct genotype profiles from different DNA samples (Demichelis *et al*., 2008). SNP array analysis was performed by the Mayo DNA Sequencing Core Facility in Rochester, MN, USA. The relatedness of the original tumor and ascites grown in corresponding PDX model was determined by comparing the SNP profiles of the respective samples.

### 2.5 Tissue processing and immunohistochemistry

Hematoxylin and eosin (H&E) staining and immunohistochemistry (IHC) were performed on formalin‐fixed, paraffin‐embedded (FFPE) tumor tissue from sacrificed mice or on cells from ascites pellets. Ascites liquid was collected at sacrifice, spun for 10 min at 670 ***g***, and the cell pellet was spread on glass slides, fixed, and stained. Antibodies used: NRG (ThermoFisher PA5‐13204; 1 : 00), AKT (Cell Signaling 9272; 1 : 250), phosphorylated AKT (Novocastra NCL‐L‐AKT; 1 : 250), mTOR (Abcam Ab32028, Cambridge, MA, USA; 1 : 1000), EGFR (Dako 5207, Carpinteria, CA, USA), Her2 (Dako K1494). Slides were imaged using a Nikon Eclipse 50i microscope and a Nikon PS Ri2 camera.

### 2.6 Immunoblotting

Tissue and cells for protein analysis were prepared as previously described (Zhang *et al*., 2018). Briefly, tissue or cells were lysed in NTEN buffer, and supplemented with complete protease inhibitor cocktail (Roche 11836170001, Indianapolis, IN, USA) and phosphatase inhibitor cocktail II (Boston BioProducts BP‐480, Boston, MA, USA). Protein concentration was determined using the Pierce BCA Protein Assay Kit (Thermo Scientific 23225). Protein (30 µg) was separated by SDS/PAGE, then transferred to nitrocellulose membrane and blotted with corresponding antibodies. Densities were measured by labwork™ image acquisition analysis Software, and the relative expression levels plotted.

### 2.7 Quantitative polymerase chain reaction

Complementary DNA was synthesized using 275 ng of total RNA, Random Hexamers (Invitrogen, cat N8080127, Carlsbad, CA, USA) and Superscript III [Invitrogen 18080093]. Three Taqman probes/primer sets were used to cover the gene: Hs00247620_M1, Hs00247624_M1, Hs01101538_M1 (ThermoFisher cat. 4331182, cat. 4351372). GAPDH was used as an internal control (Hs02758991_G1). qPCR was run on Applied Biosystems 7900HT using the following conditions: 40 cycles with 95 °C for 15 seconds and 60 °C for 1 minute and Taqman gene expression master mix (ThermoFisher 4369016). The Δ*C*_t_ from GAPDH was calculated and plotted as absolute value. Cases where NRG1 was undetected were given a Δ*C*_t_ of 0.

### 2.8 Statistical analysis

All data were presented as mean ± SD. The mean was the average of at least triplicate samples in each experiment. Each experiment was repeated at least three times. Student's *t*‐test was used to analyze the results. Differences were considered to be statistically significant at *P* < 0.05.

## 3 Results

### 3.1 Molecular characterization of ovarian tumors using MPseq

Patient‐derived xenografts in conjunction with next‐generation sequencing technologies provide a great system for identification of potential therapeutic targets and testing clinical activities of corresponding drugs *in vivo*. Using a whole‐genome MPseq protocol (Gaitatzes *et al*., 2018) we profiled three HGS‐OC tumors to identify CNV and gene fusions, and studied molecular responses of genomically chosen therapies in associated PDX models (Fig. 1A). In this study we specifically focused on tumors with structural alterations involving genes of the ERBB pathway, comparing responses to anti‐HER therapy alone and in combination with standard chemotherapy.

Mate‐pair next generation sequencing analyses of a primary tumor from the patient, as well as its matching PDX model, designated as PH026, revealed very similar profiles in the landscape of structural alterations and CNV between the two (Fig. 1B,C), consistent with previous studies reporting molecular closeness of original tumors to their PDX derivatives of early passages (Weroha *et al*., 2014). As expected for high grade serous subtype tumors, multiple gains (blue lines) and deletions (red lines) were found, indicating high levels of genomic instability (Fig. 1B,C). Major chromothriptic events (Jones and Jallepalli, 2012; Kovtun *et al*., 2015; Smadbeck *et al*., 2018; Stephens *et al*., 2011) involving chromosomes 11, 19, and 22, as well as a cluster of alterations on chromosome 9 were observed in both the original patient tumor and the tumor from the mouse. Additional rearrangements were identified only in tumor from the PDX. For example, three new alterations on chromosome 7q, which were not seen in the original tumor, were detected (Fig. 1C). In addition, careful comparison revealed several additional alterations within chromothripsis on chromosomes 11 and 19 in the PDX PH026 tumor. The differences between the original tumor and its derivative propagated in the PDX suggest that the tumor has evolved (Nik‐Zainal *et al*., 2012) over three generations of mice by acquiring additional aberrations. In fact, sites of chromothripsis were suggested to be particularly unstable and prone to more breakage.

**Figure 2:**
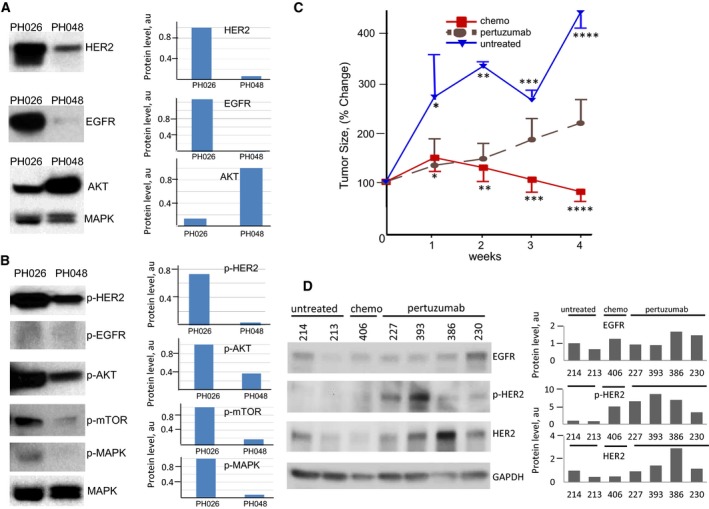
Treatment response in PH026 tumor harboring duplication of ERBB2 gene. (A) Immunoblotting analysis of the indicated proteins in two OC PDX models (designations are shown). (B) Immunoblotting analysis of levels of phosphorylation for proteins shown in (A). MAPK was used as a loading control to normalize the protein level, shown on the right panel of (A) and (B). (C) PH026 tumor treatment responses to indicated regimens. Relative tumor volumes (as percentage of initial volume at the start of the treatment) are displayed as mean ± SD. * *P* = 0.0214, ** *P* = 0.0004, *** and **** *P* = 0.00001. Student's *t*‐test was used to compare the differences. (D) Immunoblotting analysis of levels of indicated HER receptors in tumors of PH026 mice untreated or treated with chemotherapy or pertuzumab (PZ). The numbers indicate the animal IDs. Quantification of protein levels, normalized to GAPDH level, is shown on the right.

The locus containing the ERBB2 gene on chromosome 17 in the PH026 tumors also showed a number of alterations present in both the original tumor and the PDX (Fig. 1B,C). Although there was no amplification at the ERBB2 gene, a duplication supported by a number of associated reads was revealed. A high level (relative to another ovarian tumor) of HER2 expression and a high level of phosphorylation were found (Fig. 2A). Assessment of the total levels of proteins including ERBB receptors and downstream targets (Fig. 2A) and their phosphorylation status (Fig. 2B) suggested activation of the pathway. In fact, the levels of phosphorylated (p‐) forms of AKT, mTOR, and MAPK were 3‐ to 10‐fold higher in the PH026 tumor (Fig. 2B). In contrast, there was no phosphorylation detected in EGFR, suggesting that it was not involved in the signaling of this tumor.

### 3.2 Single agent anti‐HER2 therapy

To test whether blocking the ERBB2 pathway *in vivo* could provide a benefit, PH026 mice were randomized to three arms: (a) untreated control, (b) treated with PZ or (c) treated with standard chemotherapy consisting of a carboplatin/paclitaxel combination (Fig. 1A). Mice in the treatment arms received corresponding therapy for 4 weeks, and tumor volume was assessed weekly. No adverse effects such as acute weight loss or poor body condition due to either treatment were noted. The tumor volume as assessed by ultrasound in the untreated group increased 4‐ to 4.5‐fold over the 4 weeks of observation time. In the group treated with PZ, significant (*P* < 0.00001 by week 4) inhibition of tumor growth was observed compared with untreated mice (Fig. 2C). However, no tumor regression was found. In contrast, chemotherapy showed some therapeutic benefit, with a nearly 25% reduction in tumor volume as compared with initial tumor size. The regression was modest compared with what would be expected for a chemo‐sensitive tumor, but nevertheless constituted a response to chemotherapy in contrast to the response observed in the original patient, whose tumor was classified as chemo‐resistant.

Changes on a molecular level to the PDX tumors were evaluated using immunoblotting (Fig. 2D) and IHC (Fig. S1) following the prescribed treatments. Compared to the levels observed in untreated or mice treated with chemotherapy, total HER2 and pHER2 were elevated in tumors from mice which received PZ (Fig. 2D). This was surprising as rather decrease in phosphorylation upon inhibition with PZ was expected. The level of EGFR did not differ between treated and untreated tumors (Fig. 2D) suggesting that the signaling likely engaged HER2/HER3 dimer. The increase in HER2 phosphorylation can be explained by the action of a compensatory loop which is activated in response to inhibition. Together these results suggested that anti‐HER therapy is not appropriate as a first line of therapy even in the case of chemo‐resistant tumors.

### 3.3 Anti‐HER2 therapy in combination with chemotherapy

We hypothesized that the modest response to PZ in PDX line PH026, despite expressing high levels of HER2 protein, was underestimated due to the fact that the tumor at the start of the treatment was too bulky (considering the tumor to body size ratio) and did not match the most common clinical scenario where debulking surgery usually precedes standard chemotherapy treatment given to eliminate residual disease (Lee *et al*., 2012; Winter *et al*., 2008) Alternatively, the benefit of HER2‐targeted therapy might be restricted to sensitization to chemotherapy when the two are given in combination. Such a mechanism was shown for breast cancer where responses to chemotherapy were much stronger in tumors with activated EGFR when followed by administration of an EGFR‐targeting drug (Lee *et al*., 2012). To test second possibility we next randomized a PDX line carrying a different human patient ovarian tumor (designated PH048) to combination treatments consisting of chemotherapy and a PZ/TZ combination which has been shown to elicit a more complete inhibition of the ERBB pathway.

**Figure 3:**
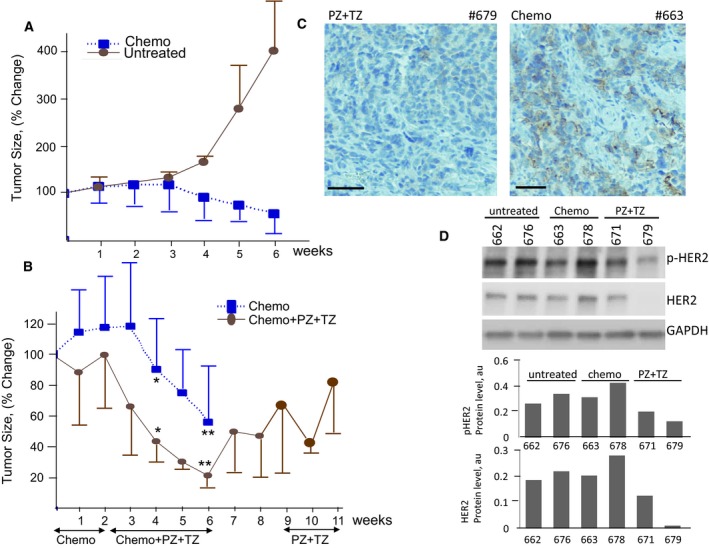
The comparison of responses to chemotherapy alone and combination with targeted therapy in PH048 tumor harboring amplification of NRG3 gene. (A) Tumor volume changes in response to chemotherapy compared with those in untreated mice. (B) Treatment responses to combination of chemotherapy and anti‐HER targeted therapy versus chemotherapy alone. The time of administration for each treatment and the duration are shown by arrows. Volumes are expressed as percentage of initial volume at the start of the treatment, as mean ± SD. * *P* = 0.02 and ** *P* = 0.04. Student's *t*‐test was used to compare the differences. (C) Immunostaining showing levels of HER2 in PH048 mice treated with targeted + chemo combination (left) and with chemotherapy only (right). Scale bars: 100 μm. (D) Changes in the levels of HER2 and pHER2 elicited by each treatment as determined by **i**mmunoblotting analysis**.** The protein levels are normalized to GAPDH level.

Mate‐pair next generation sequencing of the original human PH048 tumor showed aneuploidy with single copies of chromosomes 14, 15, 17 and 18 missing and genomic instability as well (Fig. S2). Detailed analyses of structural alterations and CNVs of this tumor revealed a gain at the NRG3 gene, a ligand for ERBB4 and ERBB3 receptors, that was previously shown to play a role in ERBB pathway activation (Liu *et al*., 2016). Genomic profiling of third generation PDX tumor derivatives of PH048 was nearly identical to the original patient's tumor (Fig. S2B). Unlike PH026 tumor, where gains rather than losses prevailed, both were evident in the PH048 tumor. No alteration at the ERBB2 gene was found (Fig. S2); however, using immunoblotting and IHC it was possible to classify this tumor as HER2‐positive (Figs 2A and S3). There was almost no phosphorylation of HER2 and downstream target proteins in PH048 tumor at the baseline (Fig. 2), suggesting lack of activation of ERBB pathway. If initial activation status was critical, we expected to have no extra benefit over chemotherapy with addition of anti‐HER2 targeted therapy. Alternatively, the baseline of phosphorylation observed in this tumor could be sufficient to promote cell growth and blocking HER2 might inhibit it. To test these possibilities PH048 mice were randomized to receive only thermotherapy or chemotherapy and PZ/TZ combination. Carboplatin/paclitaxel was given to mice in the combination arm for 2 weeks prior to addition of targeted therapy. Administration of chemotherapy alone resulted in significant reduction of tumor burden (55% median) compared to the volume at the start of the treatment (Fig. 3A). There was no difference between chemo‐treated and untreated groups in the first 3 weeks of observation. Significant (p = 0.02) difference in tumor volume between the two groups became evident at 4 weeks of treatment (Fig. 3A). In the group that received combination treatment consisting of chemo‐ with targeted therapy for four weeks after an initial 2 weeks of chemotherapy alone, an extra benefit over chemotherapy alone was observed (Fig. 3B). Specifically, at week 5 and 6 statistically significantly smaller tumors relative to baseline in the chemo‐/targeted therapy combination group were found compared to the group treated with chemotherapy only (p = 0.04). Tumor growth re‐started after treatment was stopped (weeks 7–9, Fig. 3B), suggesting that targeted therapy might have a maintenance effect. Levels of HER2 and pHER2 were compared between the groups using IHC and immunoblotting analyses. The time for harvesting tumor tissues at the end of the treatment course was 18 h after the last injection of PZ/TZ to maximize the chances to detect changes in phosphorylation level. There was, however, a notable degree of variation in the levels of total HER2 and ‐pHER2 protein among individual animals (Fig. 3D). Quantitative analysis confirmed a decrease in the level of HER2 in tumors treated with the chemotherapy +PZ/TZ combination in the majority of the mice (Fig. 3D). Similarly, the level of total HER2 protein also declined in tumors treated with PZ/TZ. Immunoblotting and IHC showed consistent results (Figs 3C, S3), thus indicating inhibition of HER2 at a molecular level. These results suggest that for chemo‐sensitive tumors expressing HER2 protein an addition of ant‐HER2 targeted therapy may improve clinical response.

**Figure 4:**
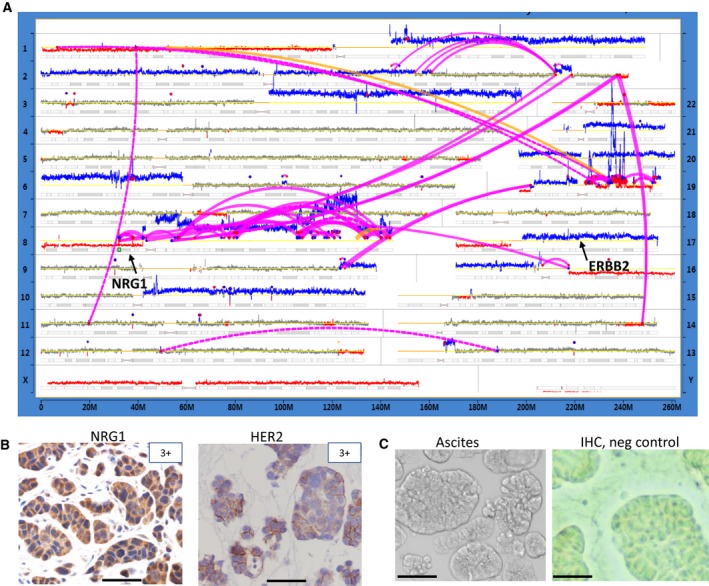
Genomic characterization of PH212 patient's tumor. (A) A genome plot showing a landscape of structural alterations in original patient's tumor. Designations are as in Fig. 1B,C. Location of NRG1 and ERBB2 genes is indicated. (B) Immunostaining showing levels (corresponding to strongly positive) of NRG1 and HER2 proteins in original patient's PH212 tumor. (C) Transmitted light image (left) and IHC image (with primary antibody omitted) of ascites (right). Scale bars: 50 μm.

We next identified an ovarian tumor, designated as PH212 that formed ascites when engrafted in mice, with no solid tumor growth. Sequencing of the original tumor revealed a major chromothriptic event on chromosome 8 (Fig. 4A) that involved the NRG1 gene, a known ligand for HER3 and HER4 receptors. Analysis of the MP reads indicates a putative fusion product involving KAT6A and NRG1 genes (Fig. S4A,B). In addition, genomic analysis showed a gain of the q‐arm of chromosome 17 (Figs 4A, S4C) that includes the ERBB2 locus. Assessment of the levels of NRG1 and HER2 by IHC showed that levels of both HER2 and NRG1 proteins were very high (Fig. 4B) compared to array of ovarian tumors tested (Fig. S5A). We hypothesized that the ERBB pathway in this tumor might be activated and targeted therapy using HER2 inhibitors could be more effective against ascites than solid tumors because of their greater accessibility for IP injected drugs. Treatment in each group started at day 7 after injection of cells, when ascites (Fig. 4C) were palpable. Mice carrying cells originating from PH212 tumors were randomized to receive chemotherapy, lapatinib or PZ/TZ alone, or a combination of chemotherapy with each targeted treatment. The response was assessed at the end of the treatment at week 5 by measuring the volume of ascites.

**Figure 5:**
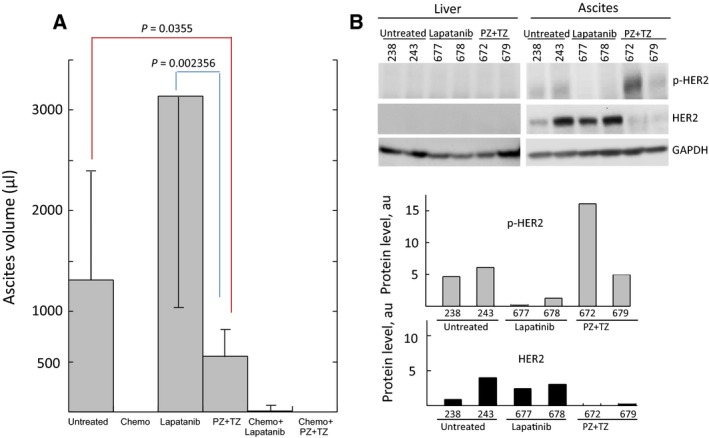
Treatment responses in ascites of PH212 model. (A) Ascites volume changes in response to targeted therapy compared with those in untreated and treated mice with chemotherapy or chemo‐ and targeted therapy combination. Volumes are expressed as mean ± SD. Student's *t*‐test was used to compare the differences. (B) The protein levels of HER2 and p‐HER2 in ascites of untreated and treated (as indicated) mice determined by immunoblotting. Quantification of protein levels normalized to GAPDH level is shown at the bottom.

No ascites were found in the groups that received chemotherapy either alone or in combination with targeted therapy (Fig. 5A), consistent with the clinical response to chemotherapy observed in this patient (chemo‐sensitive tumor). Anti‐HER2 therapy by itself was significantly better in diminishing ascites than lapatinib but inferior to the chemotherapy response (Fig. 5A). Responses in all treatment arms with the exception of lapatinib showed either reduction in tumor burden (PZ + TZ) or complete regression (chemotherapy alone or in combination with targeted therapy). Despite increases in ascites burden in untreated mice, their body weight decreased significantly (*P* = 0.0286) compared to any of the treated mice (Fig. S5B), suggesting that all of the tested treatments had positive effect on the overall condition of the mice.

To increase the chance to detect changes in phosphorylation levels of targeted proteins, mice were euthanized 6 h after their last injection. The levels of selected proteins involved in the ERBB pathway and their phosphorylation status were assessed using immunoblotting. Ascites containing tumor cells showed relatively high expression of the HER2 protein (Figs 5B and S5C) consistent with the levels observed in the original tumor (Fig. 4B). In contrast, mouse liver, which was free of metastases, did not show any expression of HER2. HER2 level in the residual ascites of mice treated with PZ/TZ was significantly diminished as compared to untreated or lapatinib‐treated mice (Fig. 5B). A similar drop in HER2 positivity was observed in ascites cells by IHC (Fig. S5C). Surprisingly, the level of phosphorylated HER2 did not decrease upon treatment with PZ/TZ (Fig. 5B).This might be due to difficulty to estimate *in vivo* how long after HER2‐targeting drug administration the tumor cells should be collected in order to detect the effect on the level of phosphorylation in the tissue because of the fast time scale of phosphorylation/dephosphorylation reactions. No phosphorylation of HER3 was detected in ether treated or untreated mice tumors (Fig. S5D), suggesting that ERBB signaling was not activated through this receptor. Phosphorylation of EGFR was notable in the ascites of mice treated with PZ/TZ (Fig. S5E), while nearly absent in untreated or lapatinib‐treated mice suggesting that the inhibition of HER2 might have triggered activation of EGFR through a feedback loop. The *in vitro* culture of the ascites collected at the end of the treatment showed significantly higher ability of cells from untreated mice to grow in dishes (Fig. S6A,B), consistent with their more aggressive phenotype, compared to counterparts from treated mice.

It was not possible to obtain specific immunostaining for NRG1 in tumor cells from ascites due to the existence of a high background of cytosolic staining of cells in suspension. We therefore utilized quantitative PCR (qPCR) with three sets of primers specific to different regions of the NRG1 transcript (Fig. S6C). Surprisingly, no expression of NRG1 in ascites samples from 2 different untreated mice (PH212 #238 and #243, Fig. S6D) was detected. In contrast, the level of NRG1 expression in tumors from PH048 mice was very high. In contrast, there was no NRG3 protein detected in PH048 (Fig. S6E), despite gene amplification found by MPseq (Fig.S2A), suggesting that NRG3 expression is down regulated and does not depend on gene copy number change. No difference in expression between the 3 probes for NRG1 (designed to detect different NRG1 isoforms) was found in any of the samples including prostate cell line BPH1 and another OC primary tumor, OvCa3 (Fig. S6D).

To confirm the matching identity of DNA from ascites of PDX mice to original PH212 human tumor, SNP fingerprinting analysis was performed and confirmed their relatedness (Fig. S7A). This result suggested that ascites cells might did not carry the alteration at the NRG1 locus or alternatively, lost expression due to gene regulation upon tumor propagation in mice.

### 3.4 Comparison of molecular makeup of original tumor to ascites of PDX

To get an insight into possible factors underlying lack of solid tumor growth in PH212 mice, molecular profiling of the tumor and the ascites was performed. Genomic alterations were identified and compared using RNAseq analysis. RNA isolated from FFPE material of the original PH212 patient’ tumor, and frozen ascites cells from associated PDX mice, was sequenced using the RNAseq Access protocol. Bioinformatics analysis was performed to compare fusion genes present in all three samples and the expression of genes within the ERBB2 pathway. A KAT6A‐NRG1 fusion identified by MP sequencing was not found in any of the other samples including FFPE original tumor (Table 1), indicating that it was not expressed. However, several highly expressed fusion products were detected by RNAseq in all three samples. These are KANSL1‐ARL17B, PSMC4‐SIPA1L3, and SUPT5H‐ACTN4. Concordance between MP and RNAseq data was 64% as 9 out of 14 putative fusion genes were seen in at least one of the samples with the RNAseq protocol. Interestingly, MP analysis showed 3 putative fusion genes within the chromothriptic event on chromosome 8: KAT6A‐GPR124, KAT6A‐NRG1 and NRG1‐GPR124. Only KAT6A‐GPR124 was also detected by RNAseq and only in the frozen ascites material. Despite the fact that both gene‐partners within the fusion showed high expression in the original FFPE patients sample (Fig. S7B), the KAT6A‐GPR124 fusion was not detected by RNAseq. This discrepancy can be explained by partial degradation of mRNA, known to occur upon fixation, processing, and storage of FFPE material, precluding identification of some of the expressed fusion genes. The absence of any alteration involving the NRG1 gene in the RNAseq dataset indicates that the MP algorithm could not predict expression of these putative fusion genes due to the complexity of the chromothriptic event at the locus (Fig. 4A). The expression of NRG1 from the intact copy of the gene, however, was detectable by IHC (Fig. 4B) in patient's tumor. Several novel alterations were identified only by RNAseq in ascites of PDX PH212 and were not seen by MP in the original tumor. Among those are MTMR3—CABP7, RYK‐AMOTL2, SIPA1‐CAPN1 detected in both frozen ascites samples with 15 or more junction spanning reads (Supporting Information). These results suggest that the tumor clone that grew in the mice evolved by acquiring new genomic changes which could account for the lack of solid tumor growth but supporting cell proliferation in ascites. Alternatively, the tumor piece implanted into the PDX mice might have consisted of different clones than that analyzed by MP sequencing. Several fusion genes including AL078471.5‐BAGE2, CCDC7‐RP11‐195O1.5, POTEE‐POTEKP were seen in one of the two frozen ascites samples (Table 1). The alterations were supported by 8, 6, and 6 junction spanning reads, respectively. Since gene‐partners in the AL078471.5‐BAGE2 and CCDC7‐RP11‐195O1.5 putative fusions are located next to each other, this raises a possibility of them being false positive because of the existence of read‐through transcripts connecting exons of two neighboring genes, a common phenomenon in human transcriptome (Communi *et al*., 2001). Similarly, the RP11‐123O10.4‐GRIP fusion detected in both ascites samples consists of two genes that are closely located in chromosome 12. This interpretation is also supported by low levels of expression of each of the individual gene partners in these fusions (Table 1). Of the fusions that were detected in one or the other ascites samples, POTEE‐POTEKP appears to be true, as its partner genes are separated by several megabases on chromosome 2.

**Table 1:** Comparison of detected gene fusions in PH212 original tumor and ascites from PDX

Sample fusion	PDX 677 RNAseq	PDX 678 RNAseq	PH212 Pt RNAseq	PH212 Pt MP
AL078471.5‐BAGE2	N/D	7.5–10	N/D	N/D
CCDC7‐CCDC7	0.3	0.6	5.2	N/D
CCDC7‐RP11‐195O1.5	N/D	0.6–2.46	N/D	N/D
Col6A3‐ATP6V1H	N/D	N/D	N/D	N/D
ERBB4‐TEX4	17.8–0.001	N/D	N/D	N/D
FAM83H‐AS1‐ASAP1	0.75–0	0.6–0	N/D	Yes
GLTSCR1‐EPS8L1	0.96–15.8	0.89–21.7	N/D	Yes
KANSL1‐ARL17B	4.6–0.19	5.1–0.49	12.9–5.2	Yes
KAT6A‐GPR124	7.3–3.16	6.84–2.9	N/D	Yes
KAT6A‐NRG1	N/D	N/D	N/D	Yes
MACF1‐NAV2	8.5–5.2	12.2–4.5	N/D	Yes
MTMR3‐CABP7	5.3–1.7	7.2–2.5	N/D	N/D
NRG1‐GPR124	N/D	N/D	N/D	Yes
POTEA‐XKR4	N/D	N/D	N/D	Yes
POTEE‐POTEKP	N/D	79.8–0.126	N/D	N/D
PSMC4‐SIPA1L3	187–5.3	119–3.6	60–10.6	Yes
RP11‐123O10.4‐GRIP1	0.2–2.32	0.004–1.33	N/D	N/D
RPS19‐LIPE‐AS1	283–0.16	154–0.18	N/D	Yes*
RRP7A‐RRP7B	3.6–0.8	N/D	N/D	N/D
RYK‐AMOTL2	15.9–20.1	11.7–17.8	N/D	N/D
SAMD8‐C10orf11	N/D	1.9–1	N/D	Yes
Slc29a3‐CDH23	N/D	N/D	N/D	Yes
SIPA1‐CAPN1	10.3–114	9.2–116	N/D	N/D
SPATS2‐WBP4	N/D	N/D	N/D	Yes
SUPT5H‐ACTN4	125–2893	116–3393	48–762	Yes
ZFPM2‐TRAPPC9	N/D	N/D	N/D	Yes

* Rearrangement that is not a predicted fusion by MP. N/D, not detected. The numbers represent the expression in RPKM for each fusion partner. PH212 Pt is original patient's tumor.

Consistent with the qPCR results, no expression of NRG1 was observed by RNAseq in the ascites of either PDX mouse tested (Figs S6D and S7B). Surprisingly, a very low NRG1 RNA expression level was detected in the original patient's tumor, contrary to the IHC staining shown in Fig. 4B. Since the tissue slices for both originated from the same paraffin embedded material, this result suggests that the detected level of expression at the RNA level was sufficient to result in the observed level of protein. On the other hand, since the sensitivity of RNAseq for FFPE samples is affected by the degree of RNA degradation, the RPKM value, and, therefore, detected expression might be underestimated.

High levels of RNA expression were observed for all ERBB receptors but EGFR (Fig. S7B). Interestingly the level of ERBB2 mRNA in the ascites of mice treated with EGFR inhibitor lapatinib was at least twice as high as in the patient's tumor. This is also consistent with the levels identified by immunoblotting (Fig. 5B) where lapatinib‐treated PDX demonstrated an induction of HER2 protein compared to mice treated with HER2 inhibitors. In contrast, levels of ERBB4 mRNA were considerably higher in the original tumor. Likewise, expression of each gene involved in the KAT6A‐GRP124 fusion was higher in the original patient's tumor than in the derivative ascites from mice. This is despite the fact that the actual fusion was not detected by RNAseq in FFPE sample (Table 1). Because it was present in the MP dataset, its absence in RNAseq dataset can be explained by partial degradation of the RNA in FFPE sample. There was no obvious difference in expression levels of ERBB3 between the three samples of PH212.

### 3.5 Comparison of molecular responses to anti‐HER therapy in three tested PDX models

**Figure 6:**
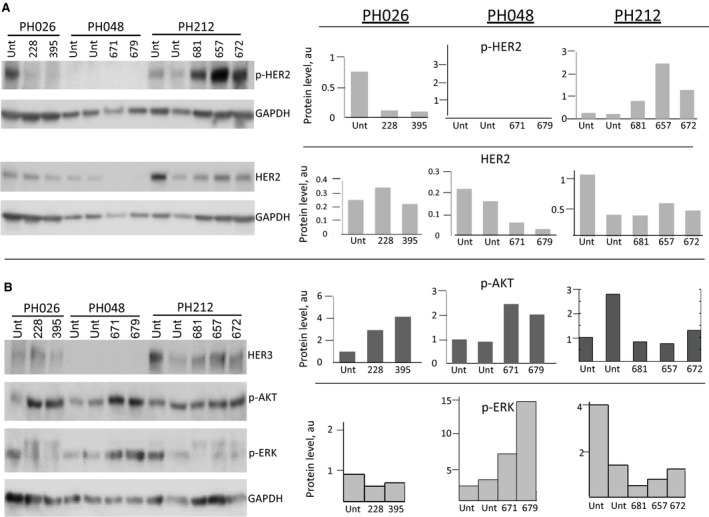
Comparison of molecular changes of ERBB pathway upon anti‐HER2 treatment in three PDX models. (A) Immunoblotting showing levels of HER2 and phospho‐HER2 of three PDX models. Quantification of protein levels, normalized to GAPDH level, is shown on the right. Unt, untreated. (B) Immunoblotting analysis of activated downstream protein targets of ERBB2 pathway. Quantification of protein levels, normalized to GAPDH level, is shown on the right.

We have compared molecular changes of targets downstream of HER2 receptors for three PDX models treated with anti‐HER2 therapies. While in all three models extra benefits were observed with the administration of HER2 inhibitors, either compared to untreated or chemotherapy only treated mice (Figs 2C, 3B and 5A), there were clear differences at the molecular level between the three. TZ and PZ are known to have distinct mechanisms of inhibition. TZ induces endocytosis of HER2, leading to its downregulation, inhibiting the ligand‐independent HER2‐HER3 action and subsequently blocking the PI3K/AKT pathway (Vu and Claret, 2012). PZ binds to the subdomain of HER2 used for dimerization, inhibiting the ligand‐dependent association of HER2 with other HER receptors (Adams *et al*., 2006), and is thus thought to be effective via the ligand‐dependent activation of HER receptors. Consistent with the reported mechanism of action of TZ, a decrease in the level of HER2 protein was observed in most of the PH048 (Figs 3D and 6A) and PH212 mice treated with PZ/TZ (Fig. 6A).This effect was absent in PH026 mice treated with PZ only (Figs 2D and 6A). In fact, the level of HER2 protein in some PH026 mice increased (Fig. 2D), suggesting cross‐activation by a feedback loop.

Total levels of HER2 protein were higher in PH212 tumors (Fig. 6A) than PH026 or PH048. While the levels of HER2 did not change considerably upon treatment with PZ in PH026, or PZ/TZ combination in PH212 mice, it diminished in PH048 and PH212 mice upon PZ/TZ combination treatment. No phosphorylated HER2 was present in tumor cells of PH048 mice, and anti‐HER2 treatment did not change it. In contrast, the level of HER2 phosphorylation decreased in the animals treated with PZ alone in PH026 and rose in PH212 mice upon treatment with the chemotherapy and PZ/TZ combination. No significant change in the level of EGFR phosphorylation upon treatment was observed in any of the PDX lines (Fig. S7C).

The heterodimer of HER2 and HER3 compared to the other HER proteins is considered the most potent (Choi *et al*., 2012) and promotes the activation of downstream PI3K/AKT and MEK/ERK pathways (Gala and Chandarlapaty, 2014) We then compared the phosphorylation status of AKT and ERK in response to anti‐HER2 therapy in all three PDX models. We did not observe a decrease in the activation of AKT or ERK (Fig. 6B). Notably, in both PH026 and PH048, mice showed an increase in AKT phosphorylation in treated mice compared to untreated, suggesting the activation of downstream targets. In the earlier study, comparison of downstream effects on ERBB pathway upon inhibition with PZ, TZ or the combination revealed that pERK signaling was inhibited by all treatments; but only PZ inhibited pAKT, suggesting their distinct action (Goltsov *et al*., 2014; Sims *et al*., 2012). In the current study, no significant inhibition of pERK was observed; in contrast, tumor cells of PH048 mice showed elevation in phosphorylation of ERK.

## 4 Discussion

Patient‐derived xenografts models have shown a great promise in modern oncology by providing a platform for testing therapies tailored toward individual tumors or subtypes. A number of studies conducted using PDX models have by and large shown PDX models to maintain original histologic, molecular and clinical properties of the donor tumor. When propagated for limited number of generations in mice, tumors show an identical to primary tumor pattern of gene amplifications and deletions gene expression (Dong *et al*., 2016; Ricci *et al*., 2014) and response to chemotherapy (Kolfschoten *et al*., 2000; Ricci *et al*., 2014; Topp *et al*., 2014; Vidal *et al*., 2012; Weroha *et al*., 2014). Assessment of tumor stem cell biomarkers also demonstrated no difference between patient's original tumors and their derivative PDX (Dong *et al*., 2016; Zhang *et al*., 2014). PDX models were shown to capture intra and inter‐tumor heterogeneity (Cassidy *et al*., 2015; Eirew *et al*., 2015; Nguyen *et al*., 2014). These key features make PDX the most faithful models for human malignancy, supporting their use in cancer research, drug discovery and preclinical development.

In this study we took advantage of large collection of serous OC biospecimens and ovarian PDXs at Mayo Clinic by profiling ovarian tumors for genomic alterations and examining responses to targeted therapy selected based on genomic findings in PDXs. HGS‐OC are known to have a high genomic instability (Malek *et al*., 2011; McBride *et al*., 2012; Patch *et al*., 2015) as well as aneuploidy (Silvestrini *et al*., 1998; Thornthwaite *et al*., 2013). Thus, in our study we focused on analyses of structural genomic alterations and CNVs (Harris *et al*., 2016) to identify potentially targetable changes using the MP sequencing protocol. Consistent with previous reports (Cybulska *et al*., 2018), we found that the landscape of genomic alterations in each tested tumor was retained in its corresponding PDX (Figs 1 and S2, Table 1). Likewise, responses to paclitaxel/carboplatin observed in patients were recapitulated in corresponding PDX mice.

Since the majority of OC patients eventually develop chemo‐resistant disease and relapse, it is critical to find alternative therapies. In several recent studies efficacy of a few small‐molecule inhibitors with cytotoxic activities were tested in PDX models for OC. For example, Vidal *et al*. reported that novel DNA minor groove binder lurbinectedin was moderately effective in inhibiting growth of PDX tumors as a single therapy and had a strong synergistic effect when combined with cisplatin, especially in the treatment of cisplatin‐resistant tumors (Vidal *et al*., 2012). In another study, antifungal agent itraconazole was shown to have increased the efficacy of paclitaxel in combination treatment of PDX models of serous adenocarcinoma and carcinosarcoma (Choi *et al*., 2017). New inhibitor of** **POLI (RNA polymerase) CX‐5461 was described to have preferential activity against cancer cells (Bywater *et al*., 2012) and its efficacy increased in taxane‐resistant OC cells (Cornelison *et al*., 2017). Other studies which examined the efficacy of targeted therapies reported their synergistic activity with chemotherapy (Brana *et al*., 2017; Groeneweg *et al*., 2014) or a maintenance effect (McCann *et al*., 2011). It is important to note that tumor growth in these studies was only inhibited compared to untreated controls and did not include a reduction in tumor volume. Indeed, a complete regression of tumors in PDX models is rarely observed, particularly in response to targeted therapies (Migliardi *et al*., 2012). These models, however, are invaluable in the preclinical testing of drug combinations. For metastatic sarcomas a correlation of 81% between drug sensitivity observed in PDX models and clinical outcome in patient‐tumor donors was noted, claiming PDX utility for personalized therapeutic decision making (Stebbing *et al*., 2014).

Coupling PDX models with high‐throughput genomic analyses of the tumor further strengthens the power of the PDX platform to study correlations between specific genomic alterations and a therapeutic response. A number of studies have reported the reproducibility and the clinical translatability of this approach (Gao *et al*., 2015; Izumchenko *et al*., 2017). So called co‐clinical trials (Hidalgo *et al*., 2014) have been conducted in which PDX models developed for patients enrolled in clinical trials were treated with the same drugs and were demonstrated to have parallel clinical responses in patients. For example, testing efficacy of EGFR inhibitor cetuximab in a set of colorectal PDX models, Bertotti *et al*. found a correlation between the clinical response and the presence of EGFR amplification (Bertotti *et al*., 2011). Additionally, whole exome sequencing of PDX models established from 92 patients with various solid tumors was performed to identify genomic changes to help guide therapeutic intervention, and found clinical outcomes to be consistent with hypotheses derived from sequencing data (Izumchenko *et al*., 2017).

In our study different alterations involving genes of the ERBB pathway (Fig. 1A) identified in three individual tumors suggested activation of this pathway. These genomic changes represented the top candidates for targeted therapeutic intervention in each tumor. We therefore tested efficacy of HER2‐targeted therapies alone and in combination with chemotherapy in hope of identifying factors which may contribute to the responses. Significance of HER2 as a driver in OC is controversial. Despite the high percent of OC cases expressing HER2 at 2+/3+ level, clinical responses to anti‐HER therapy have been very modest (Bookman *et al*., 2003; Gordon *et al*., 2006),(Makhija *et al*., 2010; Reibenwein and Krainer, 2008). Consistent with previous reports, we found high levels of HER2 protein (using IHC and immunoblotting) in all three OC tested (Figs 2, 3, 4) regardless of genomic alteration presence. Targeting HER2 as a single therapy consistently resulted in tumor growth inhibition compared to untreated mice. The fact that the response was inferior to that of chemotherapy, which in the PDX model for ascites resulted in the complete killing of tumor cells, suggested that it is unlikely to be effective in a clinical setting as a single therapy.

Synergistic antitumor activity of PZ/TZ combination therapy in OC xenograft model *in vivo* was reported earlier. Comparison of molecular responses to PZ, TZ or the combination of the two revealed both common and distinct downstream effects, suggesting that complementary pathways might be involved (Sims *et al*., 2012). In a later study, the synergy was proposed to be due to independence of the combination effect on HER3/HER2 composition (Goltsov *et al*., 2014). Specifically, this report showed that PZ/TZ combination treatment caused an increase in the level of HER3 receptor which in turn, reprogrammed the ERBB pathway changing HER2 homodimerization to HER3/HER2 heterodimerization. Investigation of mechanisms underlying synergistic effects of PZ/TZ combination led to identification of a novel pathway. This treatment was demonstrated to cause inhibition function of NRF2 transcription factor and a subsequent repression of NRF2‐dependent antioxidant response pathway in ovarian cancer cells with moderate and high expression of HER2 (Khalil *et al*., 2016b). Moreover, treatment with PZ/TZ affected transcription of NRF2 itself causing methylation of its promoter and subsequent gene silencing (Khalil *et al*., 2016a). Pre‐activation of NRF2, on the other hand, was shown to attenuate the combined growth inhibitory effects of HER2‐targeting drugs PZ and TZ (Khalil *et al*., 2016b). In our study, the addition of PZ/TZ to chemotherapy resulted in an extra benefit, causing greater tumor regression of the chemosensitive PH048 tumor (Fig. 3). Similar to an earlier report (McCann *et al*., 2011), targeted therapy in this case also had a maintenance effect after cessation of chemotherapy.

Collectively, these data indicate that blocking HER2 in HGS‐OC can sensitize tumors to chemotherapy. Such a phenomenon was first described for chemo‐targeted combination therapy in triple negative breast cancer (Lee *et al*., 2012).

It was of a particular interest to compare responses to therapies in PH212, the PDX model of ascites, with those PDX with engrafted solid tumors which had not produced ascites (PH026 and PH048). It remains unclear why no solid tumors were formed in the PH212 model. We predicted that the efficacy of IP‐injected drugs might be better in this model because of better accessibility of cells to the drugs. However, this was only observed with the administration of chemotherapy that resulted in a complete response in the PDX PH212 ascites model. Neither lapatinib nor PZ/TZ combination elicited such a response. Since no tumor cells were left at the end of each combo treatment that included chemotherapy, it was not possible to assess whether PZ/TZ or lapatinib provided extra benefit when administered together with chemotherapy. The levels of HER2 were high in all three tumors tested and changes in phosphorylation were noted upon targeted treatments (Figs 3, 5 and 6). The levels of possible HER2 dimer partners, EGFR and HER3, were generally low or in some cases absent, with the phosphorylation level often undetected. Analyses did not provide a clear indication of which of the two was engaged in promoting HER2 downstream effects. It appeared that in PH048 this was EGFR1, whereas in PH026 and PH212 this likely included both. The molecular analyses of responses *in vivo* proved to be challenging, as the timing of phosphorylation is hard to determine. Nevertheless, in one model, PH026, the decrease in pHER2 level upon treatment with PZ was consistently observed (Figs 1C and 6A).

Although most DNA alterations found in patient tumor PH212 were detected in the derivative PDX, the expression of a key player in ERBB signaling pathway, NRG1 was lost in the PDX completely, whereas it was very high in the original tumor (Fig 5B, Figs S6 and S7). This loss might be due to adaptive changes occurring in ascites to enable the cells to grow in suspension.

## 5 Conclusions

The targeting of presumably activated ERBB2 pathway components alone in HGS‐OC results in a modest treatment benefit. However, a combinational therapy including both chemotherapy drugs and HER2 inhibitors provides a far better response. Further studies are needed to address the development of recurrence and the sensitivity of recurrent disease to these treatments. Genomic information obtained from the original tumor greatly helps therapy decisions, which can be faithfully tested in PDX models. However, additional analyses might be needed in each individual case to confirm the status of the target at the protein level.

## Conflict of interest

The authors declare no conflict of interest.

## Author contributions

IVK conceived and designed the project. FRH, PZ and LY performed treatment experiments and interpreted the results. PZ and LY performed molecular characterization of the tumors and analyzed the data. XH oversaw PDX development and maintenance. GV provided bioinformatics analyses of mate‐pair sequencing results. KL and SJW provided clinical information and helped with the data interpretation. SJW and IVK provided study supervision. IK wrote the manuscript. All authors contributed to the review and the revision of the manuscript.

## Supporting information


**Fig. S1.** IHC staining for HER2 and EGFR proteins in PH026 model.

**Fig. S2.** Genome plots showing landscape of structural alterations in donor tumor and PDX tumor.

**Fig. S3.** IHC staining for HER2 and EGFR proteins in PH048 model.

**Fig. S4.** Alterations at NRG1 and ERBB2 genes in PH212 model.

**Fig. S5.** Immunostaining for NRG1 and HER2 in patient tissues and mouse ascites.

**Fig. S6.** Expression level of NRG1 in ascites detected by qPCR. Immunoblotting for NRG3.

**Fig. S7.** Expression level of ERBB pathway genes in PH212 model determined by RNAseq.

**Data S1.** RNAseq supporting reads for gene fusions in PH212 tumors.
