# Supplementary figures and images for: Targeting HER2 in patient‐derived xenograft ovarian cancer models sensitizes tumors to chemotherapy
Source: Mol Oncol. 2018 Dec 21;13(2):132–52. doi: 10.1002/1878-0261.12414 (PMC6360362; doi:10.1002/1878-0261.12414)

Breast cancer

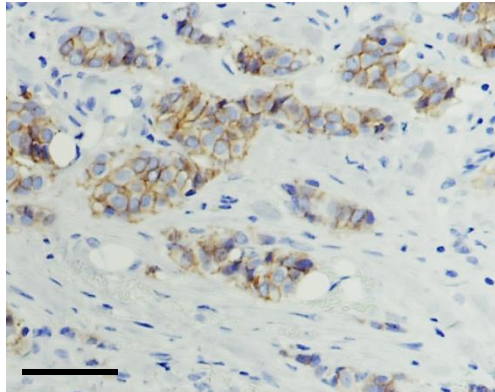

HER2

Chemo

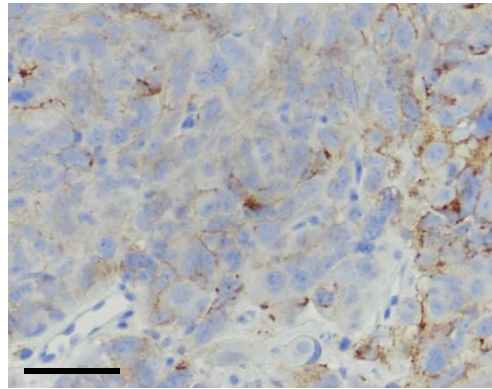

HER2

Chemo

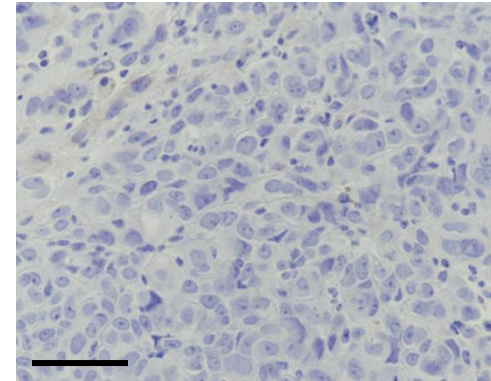

EGFR

PH026

Supplement: Supplementary file 1 — Fig. S1. IHC staining for HER2 and EGFR proteins in PH026 model. [file MOL2-13-132-s001.pdf]

**A**

PH048, patient's tumor

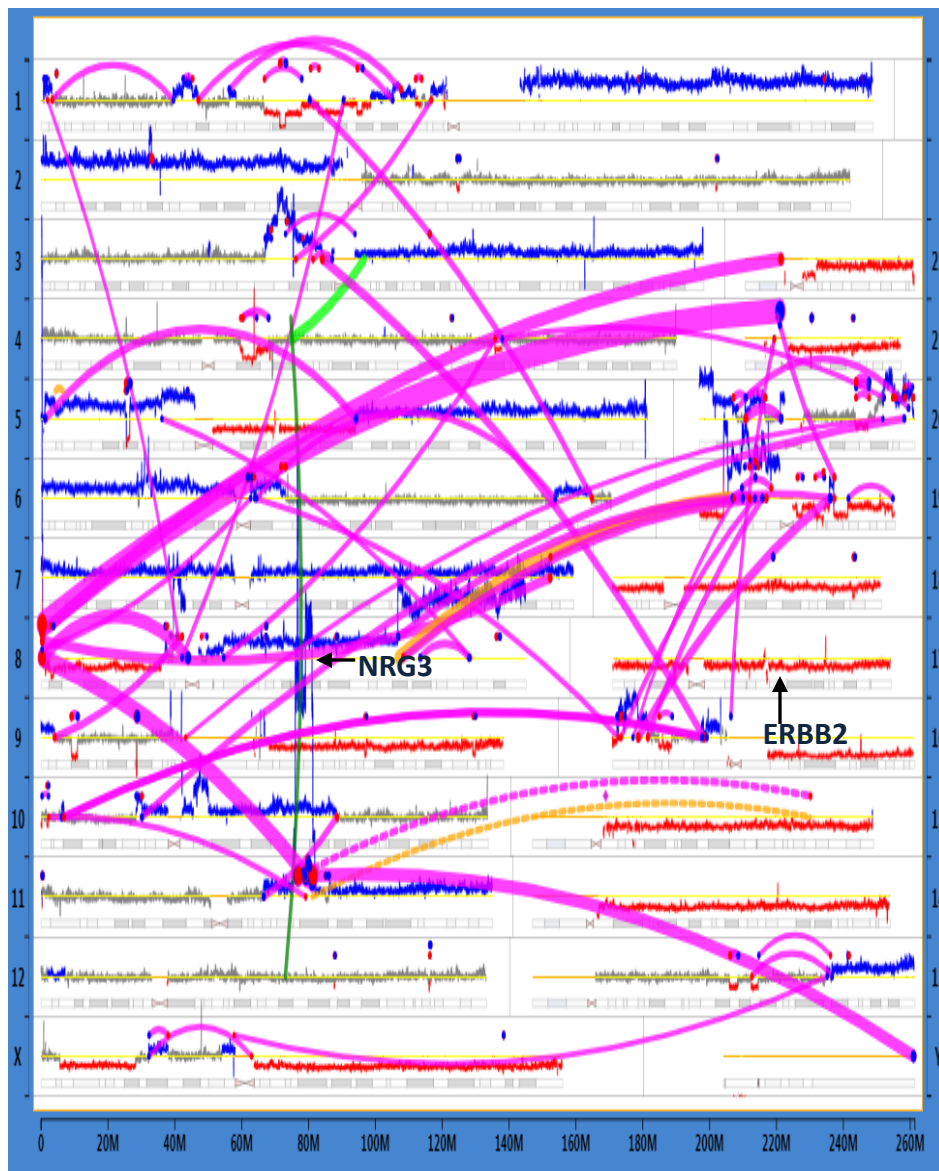**B**

PH048, PDX

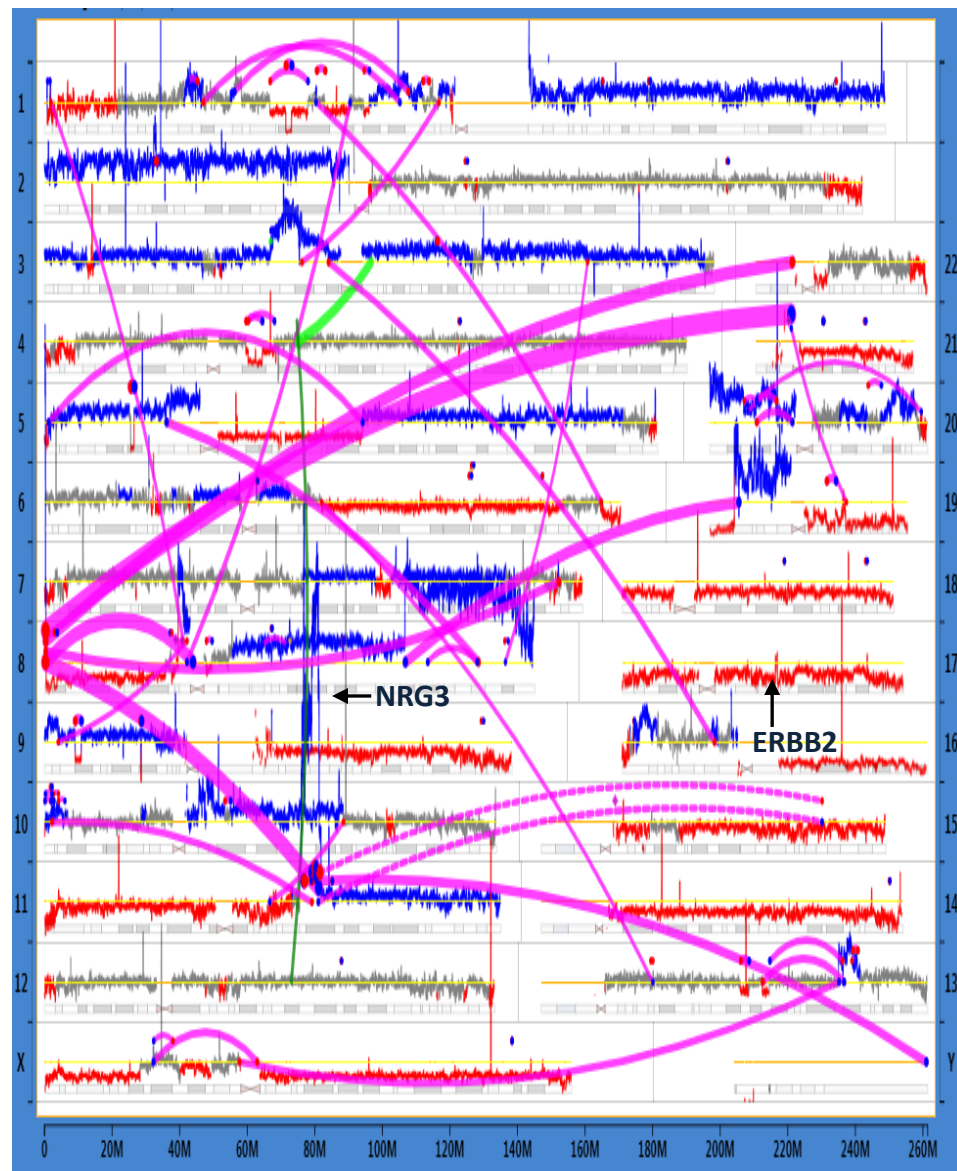

Supplement: Supplementary file 2 — Fig. S2. Genome plots showing landscape of structural alterations in donor tumor and PDX tumor. [file MOL2-13-132-s002.pdf]

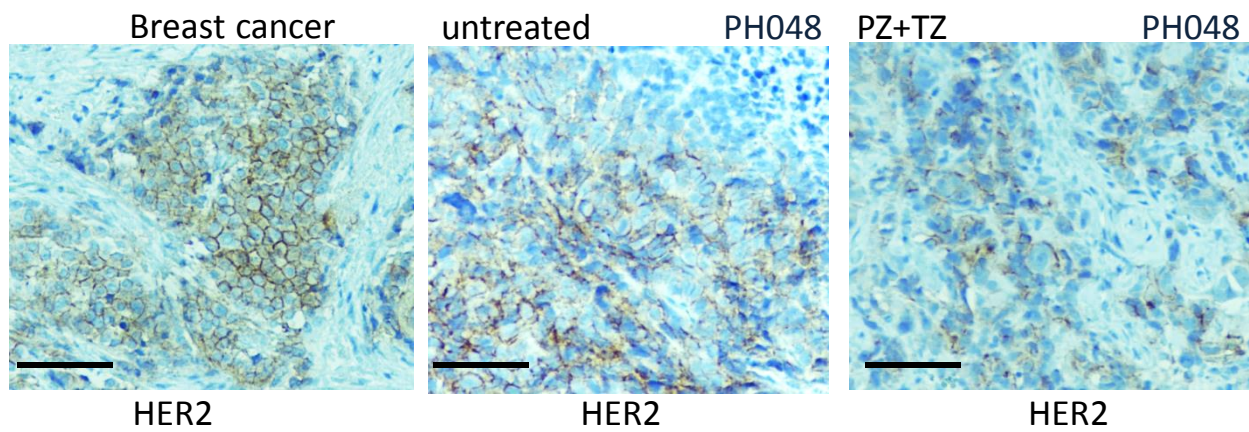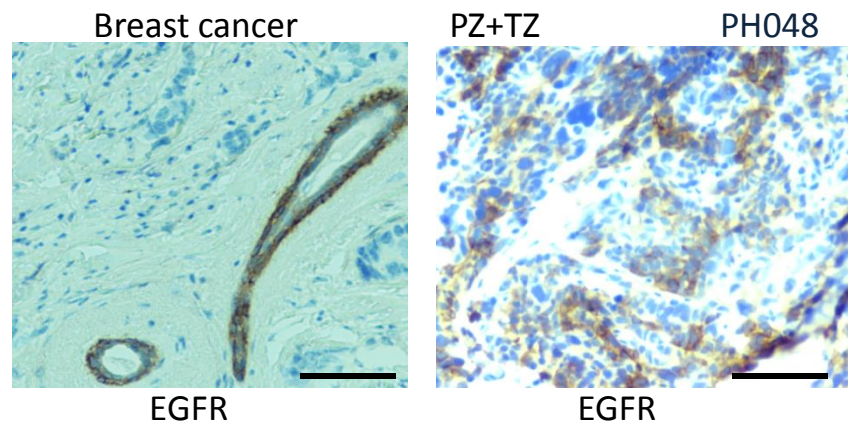

Supplement: Supplementary file 3 — Fig. S3. IHC staining for HER2 and EGFR proteins in PH048 model. [file MOL2-13-132-s003.pdf]

A

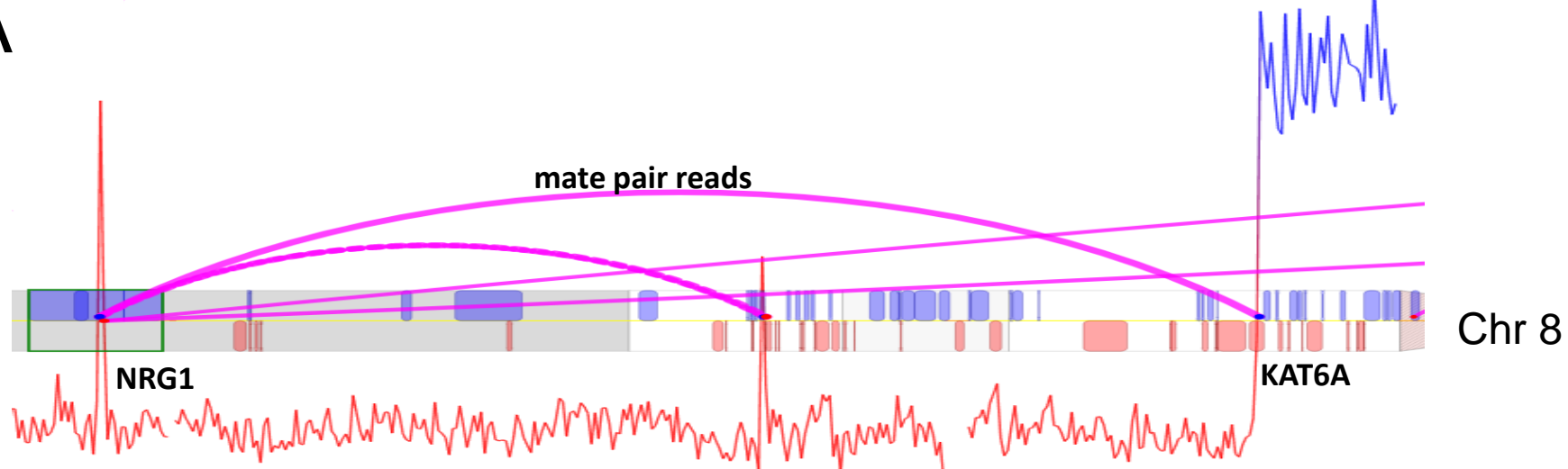

B

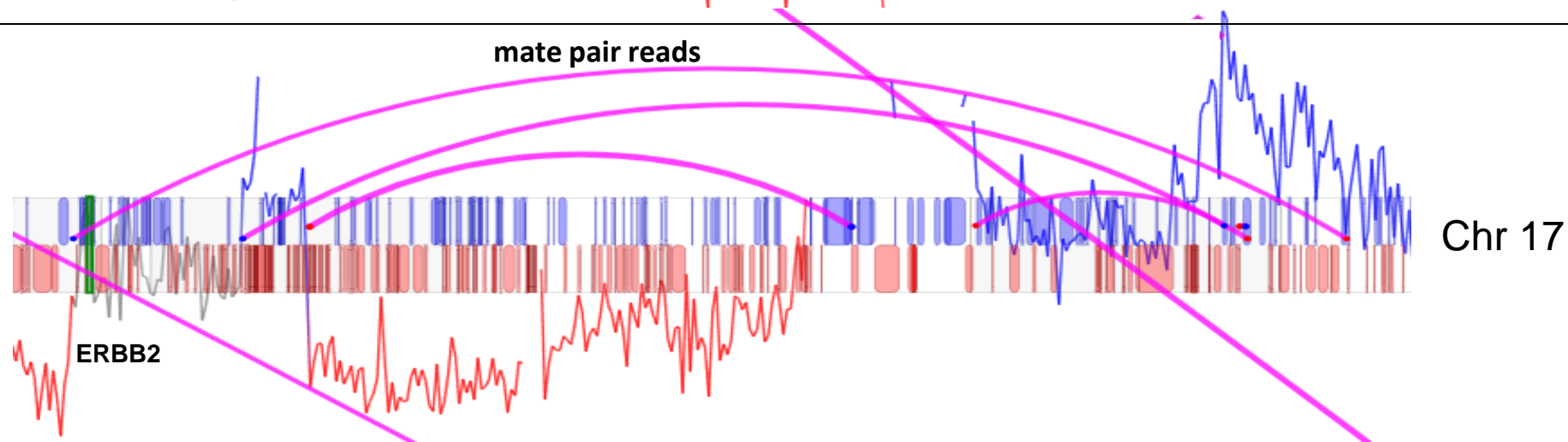

C

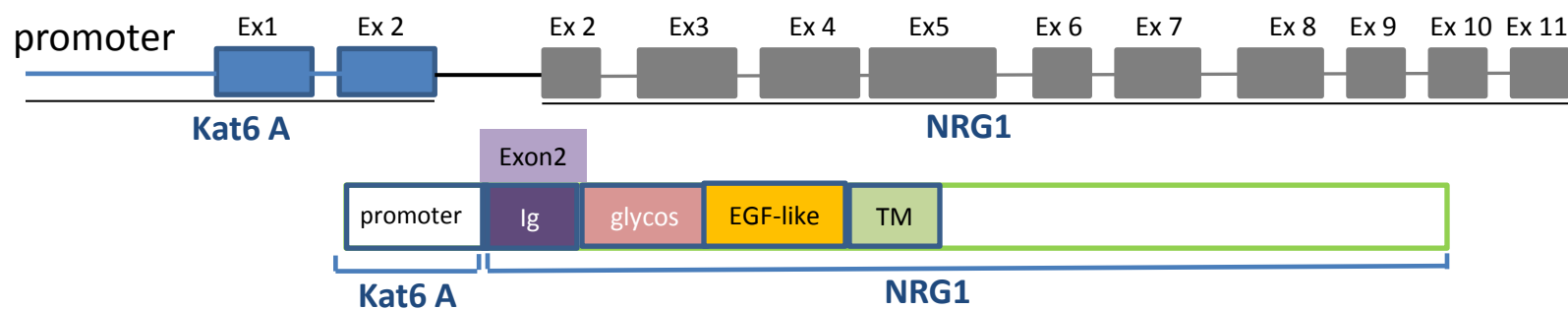

Supplement: Supplementary file 4 — Fig. S4. Alterations at NRG1 and ERBB2 genes in PH212 model. [file MOL2-13-132-s004.pdf]

**A**

Untreated

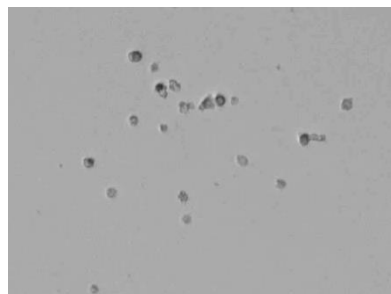

Lapatinib

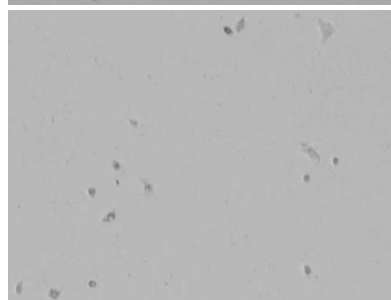

PZ+TZ

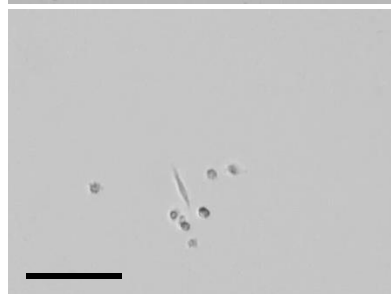**B**Cell attachment/ growth in vitro,  
# per field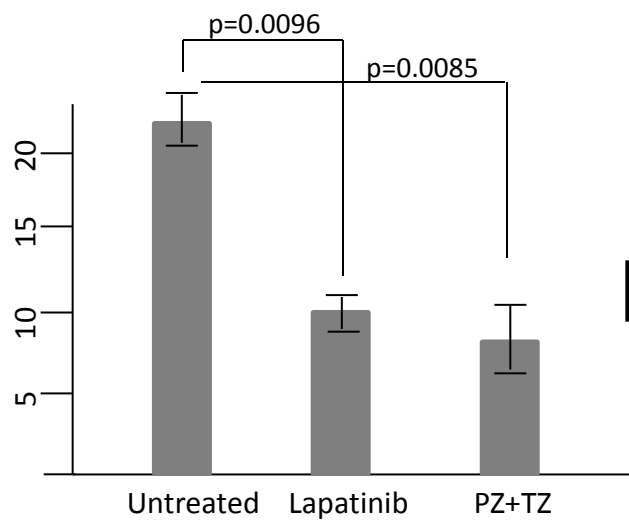**C**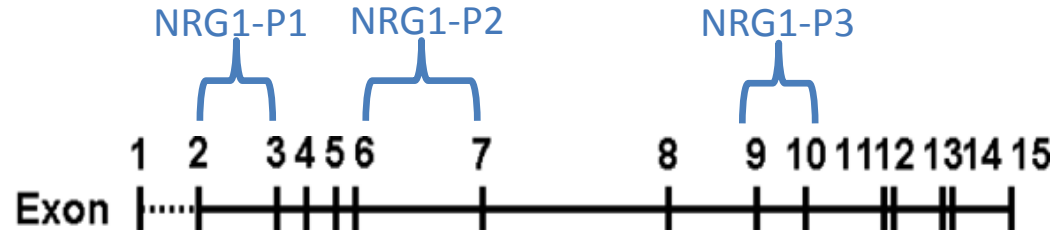**D**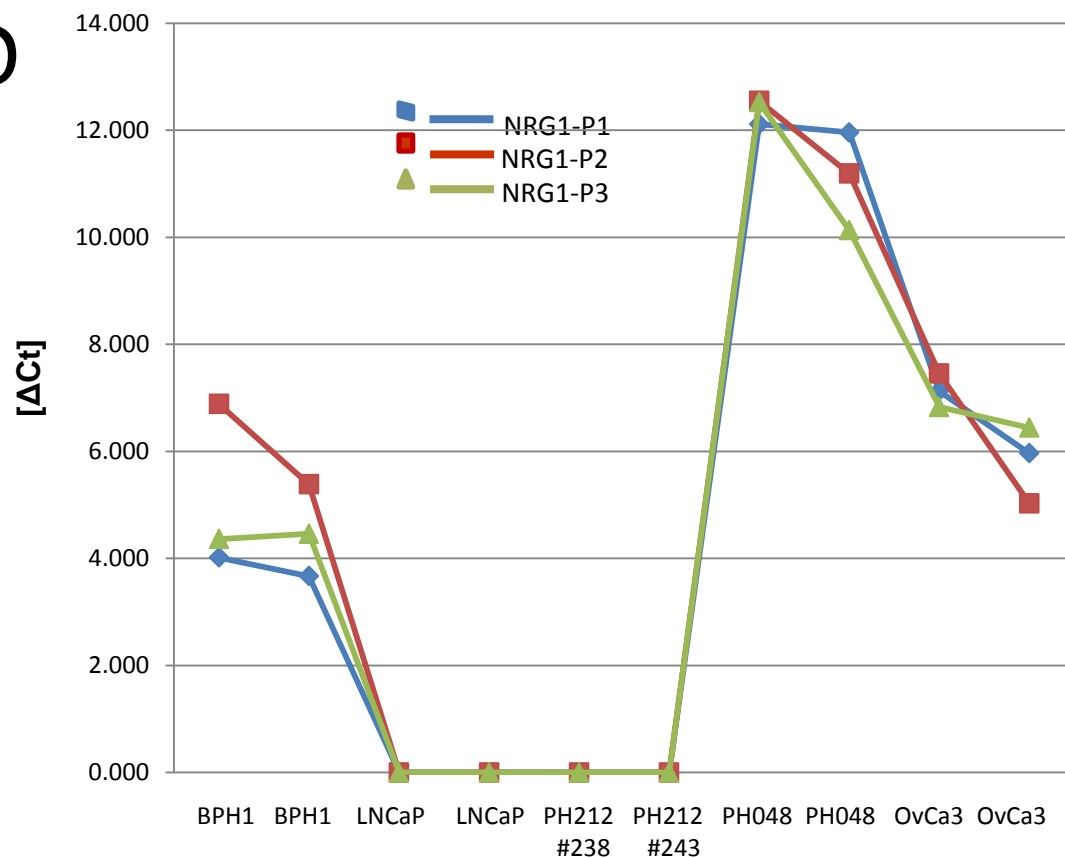**E**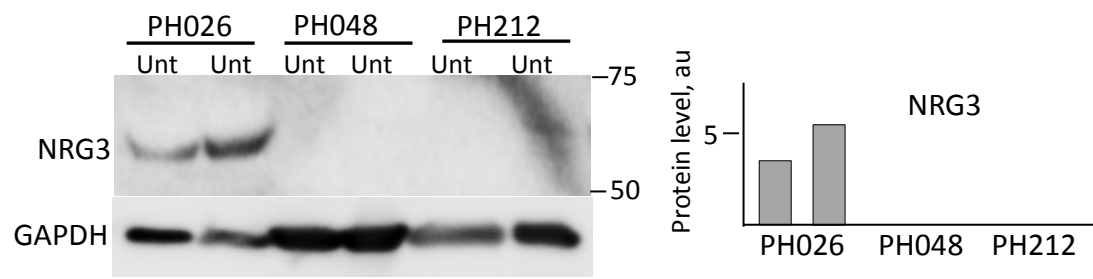

Supplement: Supplementary file 6 — Fig. S6. Expression level of NRG1 in ascites detected by qPCR. Immunoblotting for NRG3. [file MOL2-13-132-s006.pdf]
