# Supplementary material for: Targeting HER2 in patient‐derived xenograft ovarian cancer models sensitizes tumors to chemotherapy
Source: Mol Oncol. 2018 Dec 21;13(2):132–52. doi: 10.1002/1878-0261.12414 (PMC6360362; doi:10.1002/1878-0261.12414)
Supplement: Supplementary file 5 — Fig. S5. Immunostaining for NRG1 and HER2 in patient tissues and mouse ascites. [file MOL2-13-132-s005.pdf]

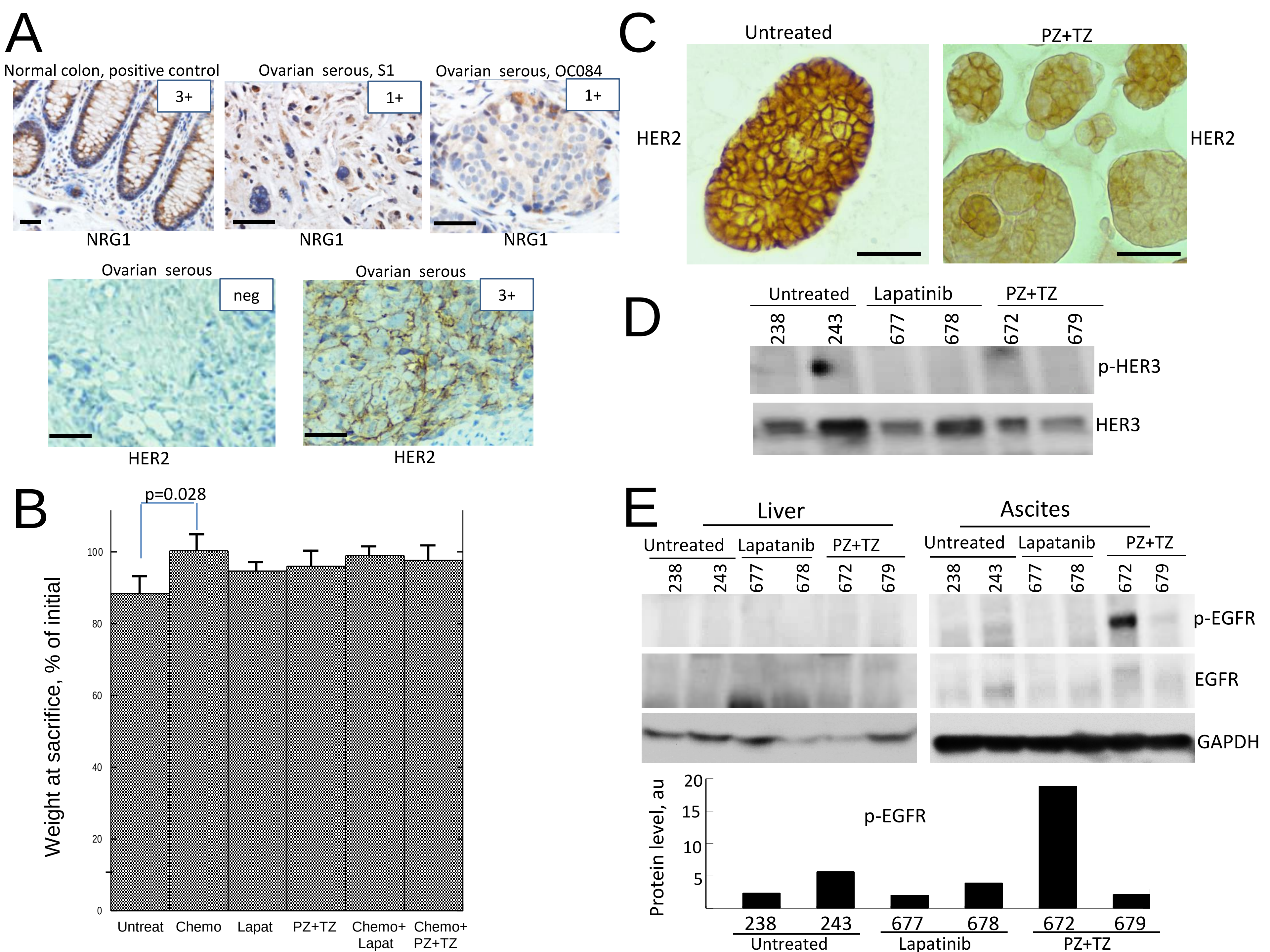

A
Normal colon, positive control
3+
NRG1
Ovarian serous, S1
1+
NRG1
Ovarian serous, OC084
1+
NRG1
Ovarian serous
neg
HER2
Ovarian serous
3+
HER2
B
p=0.028
Weight at sacrifice, % of initial
0
20
40
60
80
100
Untreat
Chemo
Lapat
PZ+TZ
Chemo+ Lapat
Chemo+ PZ+TZ
C
Untreated
PZ+TZ
HER2
HER2
D
Untreated
Lapatinib
PZ+TZ
238
243
677
678
672
679
p-HER3
HER3
E
Liver
Ascites
Untreated
Lapatinib
PZ+TZ
238
243
677
678
672
679
p-EGFR
EGFR
GAPDH
Protein level, au
p-EGFR
5
10
15
20
238
243
677
678
672
679
Untreated
Lapatinib
PZ+TZ
