# Supplementary material for: Targeting HER2 in patient‐derived xenograft ovarian cancer models sensitizes tumors to chemotherapy
Source: Mol Oncol. 2018 Dec 21;13(2):132–52. doi: 10.1002/1878-0261.12414 (PMC6360362; doi:10.1002/1878-0261.12414)

A.

Supplementary Table1.
DNA identity comparison

SNP	Patient's tumor	PDX ascites
AMEL1	C	C
RS10155597	A	A
RS11254413	A	A
RS1329546	A	A
RS1418843	T	T
RS1434774	Null	A
RS1460934	T	T
RS194520	T	T
RS2071310	A	A
RS2234002	A	A
RS2257212	GA	GA
RS2301788	CT	CT
RS2306331	CT	CT
RS2413761	T	T
RS2466701	A	A
RS2611797	CT	CT
RS34289137	Null	Null
RS3737983	TC	TC
RS4075325	AG	AG
RS4646626	G	G
RS4735133	G	G
RS4801433	T	T
RS5930933	T	T
RS7325513	AG	AG
RS805423	C	C
RS877761	G	G
RS931606	G	G

B.

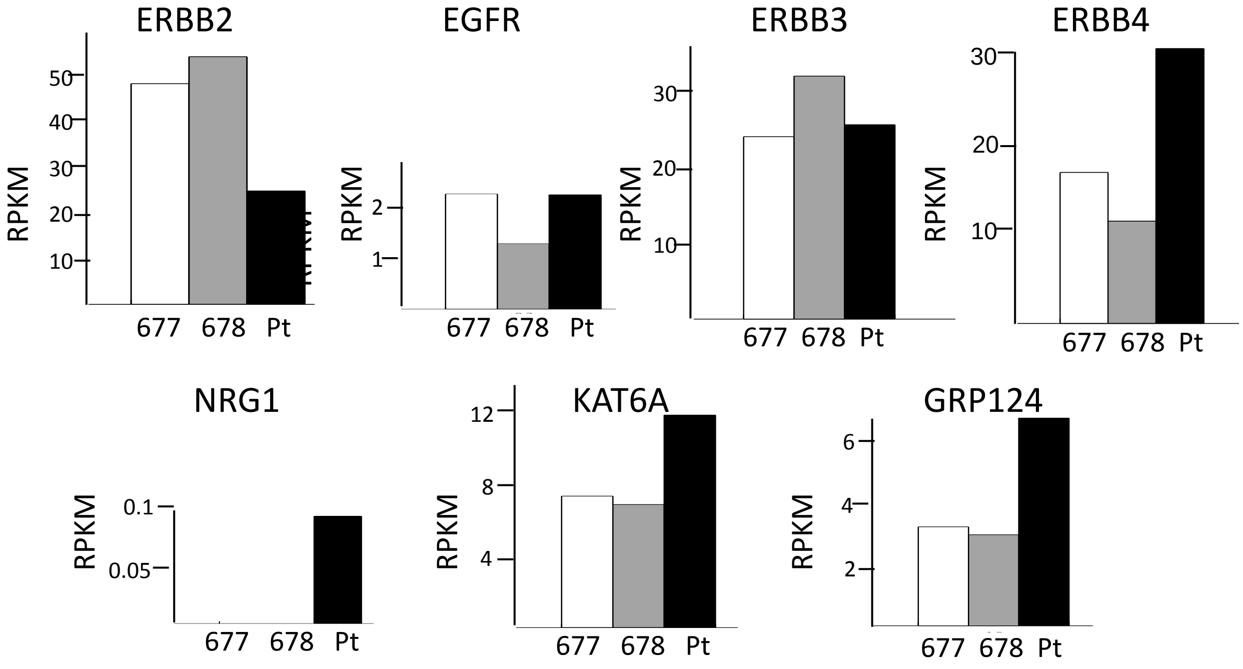

C.

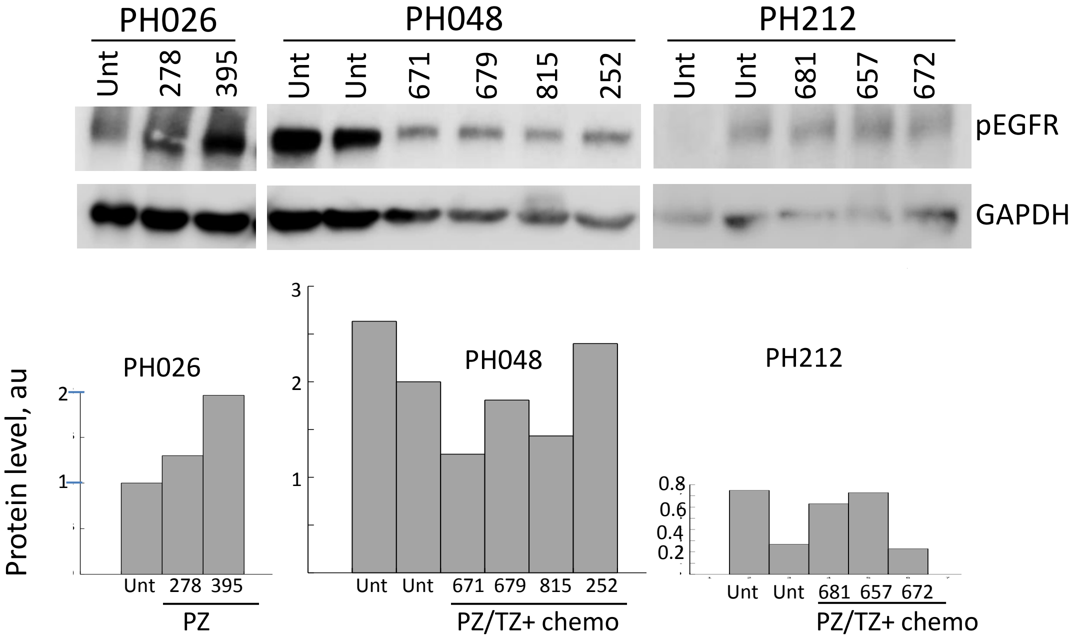

Supplement: Supplementary file 7 — Fig. S7. Expression level of ERBB pathway genes in PH212 model determined by RNAseq. [file MOL2-13-132-s007.pdf]
