# Supplementary material for: Targeting HER2 in patient‐derived xenograft ovarian cancer models sensitizes tumors to chemotherapy
Source: Mol Oncol. 2018 Dec 21;13(2):132–52. doi: 10.1002/1878-0261.12414 (PMC6360362; doi:10.1002/1878-0261.12414)
Supplement: Supplementary file 9 [file MOL2-13-132-s009.docx]

**Supplementary Figures.**

### Figure S1. IHC staining for HER2 and EGFR proteins in tumors of PH026 PDX treated with chemotherapy. Breast cancer is shown as a positive control for HER2. Scale bars = 50 μm.

**Figure S2.**  A genome plot showing landscape of structural alterations in original patient tumor PH048 **(A**) and derivative PDX **(B)**. Designations are as in Figure 1B and C. Location of NRG3 and ERBB2 genes are indicated.

**Figure S3**. IHC staining for HER2 and EGFR in tumors of PH048 PDX untreated or treated with PZ/TZ. Breast cancer is shown as a positive control for both proteins. Scale bars = 100 μm.

**Figure S4.** A close-up segment of the genome plot illustrating alterations at chromosomes 8 **(A)** and 17 **(B)** involving NRG1 and ERBB2 genes respectively in PH212 case (original patient’s tumor is shown). Blue lines correspond to gains; red losses, and magenta lines connect breakpoints at indicated positions, forming aberrant junctions. Genes are shown as blocks, red and blue colors correspond to (-) and (+) strands respectively. **C.** Schematic of putative KAT6A-NRG1 fusion as predicted by MPseq. Exons for each gene partner are shown at the top, protein domains for NRG1 included in a hybrid protein are show at the bottom.

**Figure S5**.  **(A)** IHC staining for NRG1 and HER2 proteins in series of serous ovarian cancers. Negative, low positive (1+), and strong positive (3+) protein levels are indicated. Normal colon is shown as a positive control. Scale bars = 100 μm. (**B)** The comparison of body weight changes (at the start of the treatment versus the time of sacrifice) of PH212 mice with indicated treatments. Values are shown as mean +/- SD. Student’s t-test was used to compare the differences. P value is as indicated. **(C).** IHC staining for HER2 in ascites of untreated and treated with anti-HER therapy PH212 mice. Scale bars = 50 μm. (**D)** Levels of HER3 and phospho-HER3 in ascites of PH212 PDX mice determined by immunoblotting. Treatments are as indicated. **E.** Immunoblotting showing the levels of EGFR and phospho-EGFR in lysates of liver and ascites of PH212 PDX mice. Treatments are as indicated. Quantification of protein levels, normalized to GAPDH, is shown at the bottom.

**Figure S6. (A)** Images of grown *in vitro* cells from ascites of untreated mice and mice treated with lapatinib or PZ+TZ. Scale bars = 100 μm. (**B)** Graphs showing quantification of cell growth *in vitro* (from A) as mean +/- SD. Student’s t-test was used to compare the differences. P values are as indicated. **C**. Schematic showing NRG1 gene structure with 15 exons represented as vertical bars and location of the binding for each probe designated NRG1-P1, NRG2-P2 and NRG3-P3. (**D)** NRG1 gene expression (mRNA levels) measured by qPCR and shown as delta (Δ) Ct (threshold cycle). BPH1 and LNCaP are used as negative and positive controls, respectively; PH212 #238, PH212 #243, PH048 are OC PDX tumors and OvCa3 is the original patient OC tumor. Undetected NRG1 is set to delta Ct of 0. **(E)** Immunoblotting showing the levels of NRG3 in indicated PDX models. Unt is untreated. Quantification of protein levels, normalized to GAPDH, is shown at the bottom.

**Figure S7. (A)** Table showing comparison of SNPs as determined by fingerprinting analysis in PH212 original patient tumor and PH212 PDX ascites (**B)** mRNA expression of ERBB genes in PH212 PDX mice (numerical IDs are shown) and original patient’s tumor (Pt) determined by RNAseq analyses. **(C)** Immunoblotting showing the levels of phospho-EGFR in three PDX models with indicated treatments. Unt is untreated. Quantification of protein levels, normalized to GAPDH, is shown at the bottom.
